# Activated carbon from banana peels for alizarin removal: understanding the adsorption process through isotherms, kinetics, and predictive modeling

**DOI:** 10.1186/s13065-025-01667-z

**Published:** 2025-11-12

**Authors:** Simon Bbumba, Ibrahim Karume, Ronald Kayiwa, Joan Talibawo, Phillip Musoke, Godwin Aturagaba, Moses Kigozi

**Affiliations:** 1 Department of Chemistry, College of Natural Sciences, Makerere University, P.O. Box 7062, Kampala, Uganda; 2 Department of Science, Faculty of Science and Computing, Ndejje University, P.O. Box 7088, Kampala, Uganda; 3 Department of Chemistry, Busitema University, P.O. Box 236, Tororo, Uganda; 4 Department of Physics, Busitema University, P.O. Box 236, Tororo, Uganda; 5 Department of Mechanical Engineering, College of Engineering, Design Art and Technology, Makerere University, P.O. Box 7062, Kampala, Uganda; 6 School of Public Health, Makerere University, P.O. Box 7062, Kampala, Uganda; 7 Faculty of Science, Department of Chemistry, Kabale University, P.O. Box 317, Kabale, Uganda

**Keywords:** Kinetics, Optimization, Adsorption, Modeling, Alizarin, Isotherms

## Abstract

A low-cost and efficient activated carbon was prepared from banana peels for the removal of alizarin. Non-linear kinetic studies, isotherm models, and predictive models were used to study the adsorption process. The choice of the kinetic studies and isotherm models was based on the error functions of R^2^, Adj.R^2^, chi-square, SSE and ARE. The kinetic studies using intraparticle diffusion, pseudo-first-order, Elovich, and pseudo-second-order models fit the data well. It was concluded that the pseudo-second-order model best fitted the data, thus the mechanism was by chemisorption. Isotherm studies indicated that the Langmuir, Temkin, Dubinin-Radushkevich, and Freundlich models all describe the adsorption process. The mode was best described by the Freundlich model, thus, adsorption occurred on multilayer surfaces. The quadratic model, with a high R^2^ value of 0.9740, accurately predicted the removal efficiency and identified dosage and concentration as the most significant factors. The optimized conditions were found to be 3.05 min, a pH of 5.55, a dosage of 0.014 g, and a concentration of 21.50 ppm, which resulted in a maximum removal efficiency of 92.18%. The artificial neural networks with a predictive capability (96.26%) and a correlation coefficient of 0.99999 for both training and validation sets was superior to the central composite design. This was confirmed through the comparison of the residual errors and the statistical error functions of SSE, MSE, RMSE and MAE. This study shows a dual approach of coupling activated carbon from banana peels with mathematical models to understand the adsorption process.

## Introduction

**Fig. 1:**
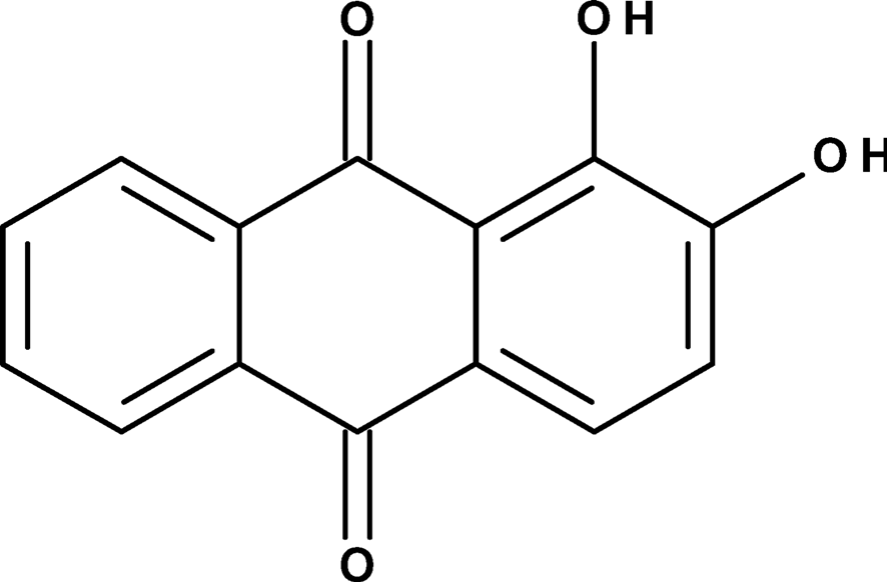
Molecular form of ALZ

Over the past decades, rapid economic growth has been observed worldwide due to the increasing population [1]. Urbanization, agriculture, and industrialization have led to an increase in the levels of toxic substances that are discharged into the water systems [2]. This has led to the contamination of both flora and fauna, thus causing widespread concern [3]. Water bodies are heavily polluted by these contaminants, and this has increased the rate of skin diseases, cancer, liver damage, hormonal imbalance, nausea, and vomiting [4, 5]. Pollution of the water bodies is a persistent problem, and over the past decade, it has been observed to have grown rapidly [6, 7]. Contaminants, including toxic heavy metals, plastics, pharmaceuticals, textile dyes, and persistent organic pollutants, are some of the commonly detected contaminants found in water sources [8, 9]. Among these, the textile dyes, which are industrial effluents, have been discharged into the water bodies, and these pose a threat to both man and aquatic life [10]. In a period of 12 months, approximately 2 million tons of dyes are applied worldwide in industries such as paper making, plastics, cosmetics, inks, and food packaging [11, 12]. Studies have shown that after the dying process, 15% of the resultant are discharged backinto the ecosystem [13]. Dyes are highly stable, persistent, and with long half-lives, which makes them exist in the ecosystem for long periods [14]. The dyes that are commonly applied include Malachite green (MG), Methyl red (MR), Methyl orange (MO), Methylene blue (MB), Alizarin (ALZ), Rhodamine b (RhB), Bromothymol blue (BMB), and Bromophenol blue (BPB) [15]. Among these, alizarin has been widely applied during several industrial processes [15, 16]. This dye is extremely resistant to degradation owing to its fused aromatic structure, as shown in Fig. 1.

The presence of the ALZ leads to depletion of dissolved oxygen, which results in less sunlight reaching the plant life underneath the water bodies [17, 18]. Its bioaccumulation in aquatic life results in the introduction of toxic chemicals to humans through ingestion [19, 20]. As a result, the scientific community has focused its research on the development of efficient dye removal techniques.

Water treatment techniques commonly used in the removal of ALZ include photocatalytic degradation, adsorption, and electrochemical degradation []. Among these techniques, adsorption has been widely used due to its simplicity in design, cost-effectiveness, simplicity in operation, wide industrial processing capacity, and ease of regeneration [23, 24]. Various adsorbents have been used in alizarin removal, such as clays, activated carbon, alumina, siliceous material, hybrid materials, and zeolites [24]. Activated carbon is widely used among these because of its large surface area, porous nature, and good thermostability [25–27]. Research has been carried out on the synthesis of activated carbon from waste biomass, particularly agricultural byproducts, for the removal of alizarin [28]. The application of waste biomass in dye removal has a positive effect on the environment as it reduces the discharged solid waste, but also offers a cheaper alternative for obtaining high-value activated carbon [29, 30]. During water treatment, the adsorbent and adsorbate have sufficient interaction with each other over a given period, thus leading to the establishment of an equilibrium between time and concentration [31, 32].

Isotherm models and kinetic studies of the adsorbate and adsorbent at equilibrium have been determined to gain insight and understanding of the adsorption process [33]. These processes are commonly modeled through the application of well-known isotherm and kinetic models [34, 35]. Isotherm models are used to determine the interaction between the adsorbate and adsorbent when the system reaches equilibrium. They further give insights into whether the process of adsorption occurs on monolayer or multilayer surfaces [36]. In addition, they can be used to qualitatively evaluate the adsorption capacity of the adsorbent [37]. It is also worth noting that the kinetic models give an understanding of the rate-limiting step but also the mechanisms of adsorption, which is either due to chemisorption or physisorption [38, 39]. The pore diffusion, film diffusion, and pore surface can be explained by the kinetic studies during the overall adsorption process [40, 41]. However, the classical method of optimization is not cheap as it involves the use of large reagents, takes a longer period and varying one parameter and keeping others constant, i.e. cannot be optimized simultaneously. Hence, the application of RSM and ANN, which saves time and the process parameters are optimized together [42, 43].

Artificial Neural Networks (ANN) and Response Surface Methodology (RSM) were used in this study [42, 44]. RSM combined with artificial neural networks enhances the model reliability and the responses generated, and this is achieved through the design of experiments and MATLAB R2024 [13]. Central composite design (CCD) was used since it does not require an experiment of the three factorial but rather a second-order quadratic model, thus giving fewer runs and is more economical. This paper presents a methodology for understanding the adsorption process through predictive modeling, utilizing CCD and ANN to analyse the impact of time, dosage, pH, and concentration on the removal of ALZ. It further elaborates isotherm models, which are used to describe the mode of adsorption, but also kinetics, which give insights into the mechanisms and rate-limiting step. Lastly, it is worth noting that no studies have been done on alizarin removal using banana peel-activated carbon coupled with isotherm, kinetics, and artificial intelligence models.

## Materials and methods

### Materials

All reagents used in this study, including sodium hydroxide, deionized water, hydrochloric acid, sulphuric acid, and Alizarin, were purchased from Sigma Aldrich. Banana peels were obtained from Kalerwe market in Kampala, Uganda.

### Preparation of the adsorbate solution

A stock solution of the dye was obtained through the dilution of 1 gram in 1 L of deionized water. Serial dilutions were prepared from the stock solution to obtain the desired concentrations, which were applied in the adsorption studies.

### Preparation of activated carbon from banana peels

Activated carbon used in this study was prepared using a synthesis method by Kigozi et al., [45] with slight modifications. Banana peels (BP) were collected from Kalerwe market around Kampala. The BP was washed and sized (~ 3 cm), then oven dried at 110 °C for 48 h. Grinding and sieving of the sample was done using a 1.0 mm sieve. A 14% wt/v H_2_SO_4_ solution was added to functionalize the powder, which was stored for 48 h, washed with water to obtain a pH of 7 and then further dried at 120 °C for 18 h. Using a 300 mL/min flow rate, 3 °C/min ramp temperature at a 2-hour holding time, the sample was activated at 600 °C.

### Adsorption experiments

Batch adsorption experiments were studied to determine the removal efficiency of the activated carbon for the alizarin dye from aqueous solution. The experiments involved 100 cm^3^ volume of Alizarin (15–45 mg/l), a dose of (0.008–0.02 g) and a pH (5–10) using NaOH and HCl. The suspensions were mixed on a magnetic shaker at 175 rpm at 20 °C, separated at a given time interval after centrifugation. A UV-3100PC Spectrophotometer calibrated in the range of 200–700 nm was used to determine the absorbance of the resultant ALZ (425 nm) [16]. The percentage removal, R (%), was determined from Eq. 1, and the adsorption capacity at equilibrium, (q_e_) was determined using Eq. 2.

1 $$\:R\:\left(\%\right)=\left(\frac{{c}_{i}-{c}_{e}}{{c}_{i}}\right)\times\:100$$

2 $$\:{q}_{e}=\left(\frac{{c}_{i}-{c}_{e}}{M}\right)\times\:v$$

Where: $\:{c}_{i}$ (mg/l) is the initial concentration of the solution, $\:{c}_{e}$ (mg/l) is the equilibrium concentration of the solution, V (L) is the solution volume, M (g) is the adsorbent mass, R (%) removal percentage, and q_e_ (mg/g) is the adsorption capacity at equilibrium.

### Isotherm models based on non-linear regression

Isotherms represent equilibrium expressions, applicable once the adsorbent and adsorbate have sufficiently interacted at a consistent temperature. The equilibrium parameters of these models often provide insights into the mechanism, the characteristics of the sorbent surface, and the level of affinity involved. Four non-linear models, that is, Langmuir, Tempkin, Freundlich, and Dubinin-Radushkevich (D-R), are shown in Table 1.

**Table 1:** Isotherm models for ALZ removal

Entry	Model	Equation
1	Freundlich	$$\:{q}_{e}$$ $$={k_f}C_{e}^{{{\raise0.7ex\hbox{$1$} \!\mathord{\left/ {\vphantom {1 n}}\right.\kern-0pt}\!\lower0.7ex\hbox{$n$}}}}$$
2	Temkin	$$\:{q}_{e}$$$$=~BIn\left( {{A_T}} \right)$$ $$+{B_T}In\left( {{C_e}} \right)$$
3	Langmuir	$\:{q}_{e}$ $=\frac{{{q_m}b{C_e}}}{{1+b{C_e}}}$
4	Dubinin-Radushkevich	$\:{q}_{e}$ $={q_d}ex{p^{\left( { - {\beta _d}{\varepsilon ^2}} \right)}}$

q_e_ is the equilibrium adsorption capacity, k_f_ is the Freundlich constant associated with adsorption capacity, and n denotes the adsorption intensity. A_T_ is the intercept derived constant, $\:{B}_{T}$ refers to the constant in the Temkin isotherm, β is the Dubinin-Radushkevich constant, ε is the Polanyi constant, and $\:{q}_{m}$ adsorption capacity at maximum

### Kinetic models based on non-linear regression for dye removal studies

Kinetic studies in adsorption play a vital role in identifying optimal conditions, clarifying the mechanism of adsorption. The prevalent models consist of Pseudo-first-order (PFO), Intraparticle diffusion, Elovich, and Pseudo-second-order (PSO), as illustrated in Table 2.

**Table 2:** Kinetic models for ALZ removal

Entry	Model	Equation
1	PFO	$\:{q}_{t}$ $={q_e}\left( {1 - {e^{\left( { - {k_1}t} \right)}}} \right)$
2	PSO	$\:{q}_{t}$ $=~\frac{{q_{e}^{2}{K_2}t}}{{1+{q_e}{K_2}t}}$
3	Intraparticle diffusion	$\:{q}_{t}$ $=~{K_{id}}{t^{0.5}}+{C_i}$
4	Elovich	$\:{q}_{t}$ $=~\frac{1}{\beta }~Ln\left( {\alpha {\beta _t}} \right)$

q_e_ is the adsorption capacity at equilibrium, while q_t_ is the adsorption capacity at a given time, K_1_ represents the rate coefficient for a first order, while t denotes the time. C_i_ represents boundary layer thickness, K_id_ denotes the diffusion constant, K_2_ refers to the rate coefficient for a second order, β indicates the constant of desorption, and α signifies the rate of adsorption

### Central composite design for modeling and optimization of process parameters

Central composite design is a non linear, multivariate model applied in the modeling and optimization of process parameters used during adsorption studies. It is further employed in regression model equations and is important in evaluating operating conditions used in experimental studies. In this study, it was used to model and optimize parameters that included concentration, pH, dosage and time. A five-level CCD was performed to determine the removal percentage (Table 3), yielding 30 runs.

**Table 3:** Process variables used in modeling and optimization

Factors	-1	0	+ 1	-α	+α
Time (min)	2.0	3.5	5.0	0.5	6.5
pH	5.5	7.0	8.5	4.0	10.0
Dosage (g)	0.008	0.012	0.016	0.004	0.02
Concentration (mg/l)	15	25	35	5	45

Table 3 shows the design points, which are Nc, 2n factorial points, and 2n axial points. The central points determine the data repeatability and experimental error. The (− 1, + 1) scale shows the independent variables. The centre has a separation distance (α) from the axial points enabling design rotation.

### Modeling with artificial neural networks

**Fig. 2:**
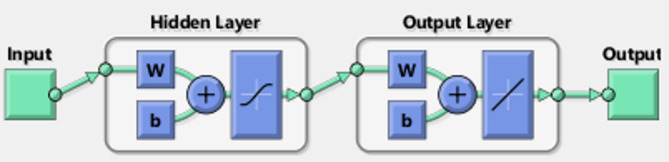
Fundamental components of ANN structure

They are efficient, non-linear analytical instruments capable of identifying relationships between both output and input data without prior knowledge of the interconnections among the involved variables. The fundamental components include the neurons, which comprise the hidden and output layers of the network (Fig. 2).

The ANN methodology tackles challenges via the implementation of a topology based on a perceptron multilayer. This MLP topology includes three neurons classified as layers, which are input, hidden, and output. The input and output variables under investigation determine the number of neurons in the hidden layer. Based on the output and input, the MLP employs a system known as training to sort and modify neuron weights according to predicted and actual data. This process delineates the relationship between input and output variables, demonstrating significant efficacy in identifying patterns within complex datasets. The backpropagation algorithm serves as an example of training algorithms, commonly employed to train artificial neural networks of varying complexities in scientific and engineering applications.

## Results and discussions

**Fig. 3:**
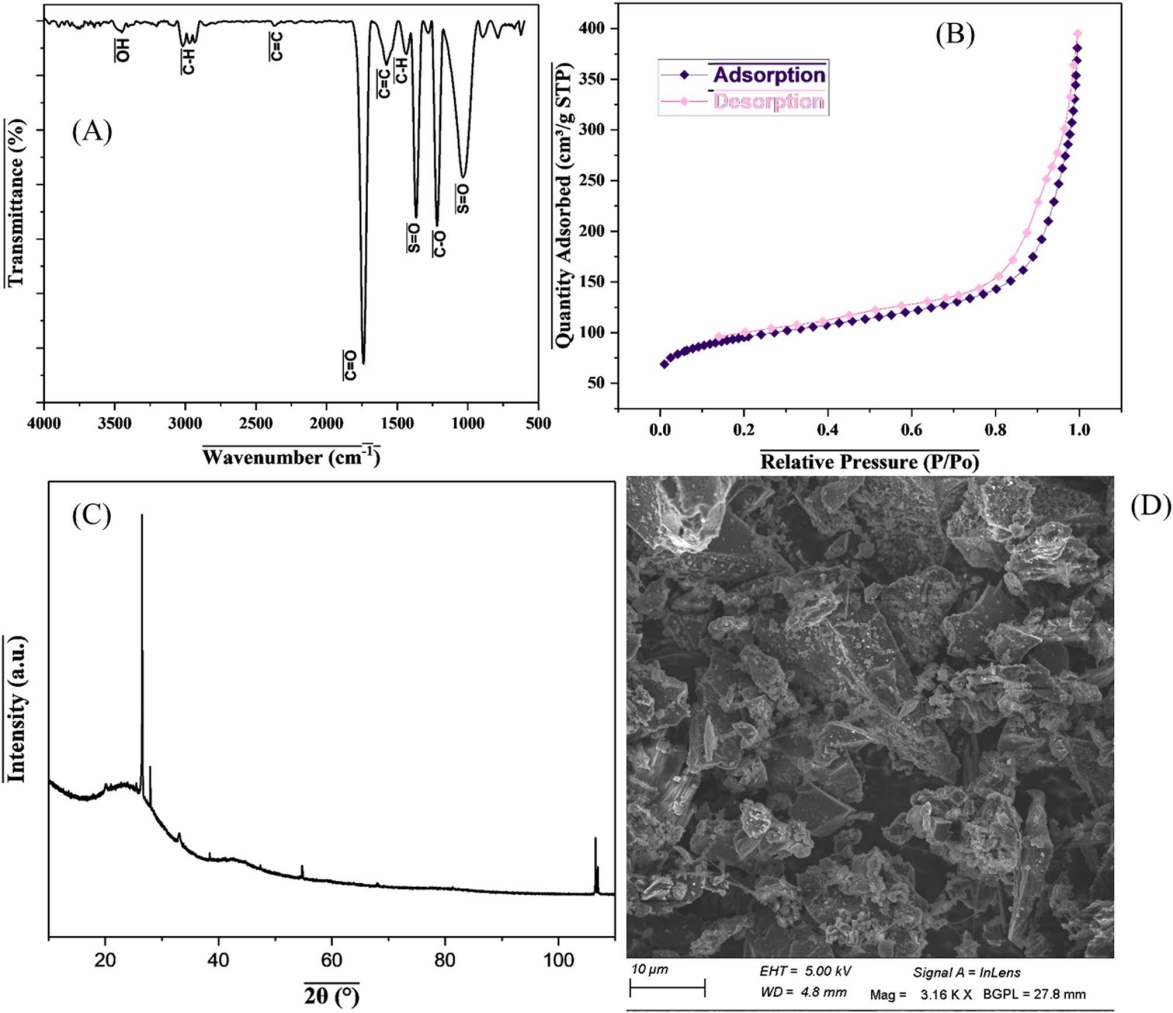
**A** FTIR spectra, **B** BET isotherm plot, **C** XRD and **D** SEM image of banana peel activated carbon

Initially, characterization was carried out on the sulphuric acid functionalized banana peel activated carbon. Fourier transform infrared spectroscopy (FTIR) analysis was carried out on the banana peel activated as shown in Fig. 3A. The sample demonstrated peaks in the fingerprint region from 1000 to 1500 cm^− 1,^ which revealed the presence of S = O, C-O, C = C, and C-H. The bands at 1740, 2350, 2976 and 3458 cm^− 1^ represent C = O, C = C, C-H, and OH functional groups, respectively. The presence of the sulphur atom shows that the activated carbon was well functionalized with sulphuric acid.

It is worth noting that the N_2_ physisorption isotherm was analyzed to determine the surface properties of the synthesized activated carbon, as shown in Fig. 3B. The mesoporous structure was confirmed by the Type IV isotherm, but the presence of micropores was evident as seen from the hysteresis loop. Table 4 further shows the porous structure of BP-AC based on adsorption/desorption studies.

**Table 4:** Surface characteristics of BP-AC as determined by N_2_ physisorption isotherm

Parameter (s)	Value
BET surface area (m^2^/g)	323.5669
Langmuir surface area (m^2^/g)	448.9595
Mean pore diameter (nm)	9.062
Total pore volume (cm^3^/g)	0.06809

In addition, the crystalline structure was investigated by X-ray diffraction, and it was shown that the material was amorphous in nature with some crystalline phase as shown in Fig. 3C. Their two distinct peaks, which are distinctive of graphitic materials which occur at 2θ ranges of 23 ° to 30 ° and 40 ° to 47 °, corresponding to the (002) and (110) crystal planes. The broad peaks at 2θ of 24 ° and 44 ° demonstrate the non-crystalline and amorphous nature of the activated carbon. Lastly, the surface morphology was investigated using scanning electron microscopy (SEM) as shown in Fig. 3D. The SEM image shows the presence of different sizes of the pores, which might be due to volatile organic matter removal during carbonization. It is also observed that the surface is rough, likely due to overactivation of BP-AC.

The mechanism, rate limiting and mode of adsorption in this study was investigated using both kinetic studies and the isotherm model as discussed in the sections below.

### Kinetic studies

**Fig. 4:**
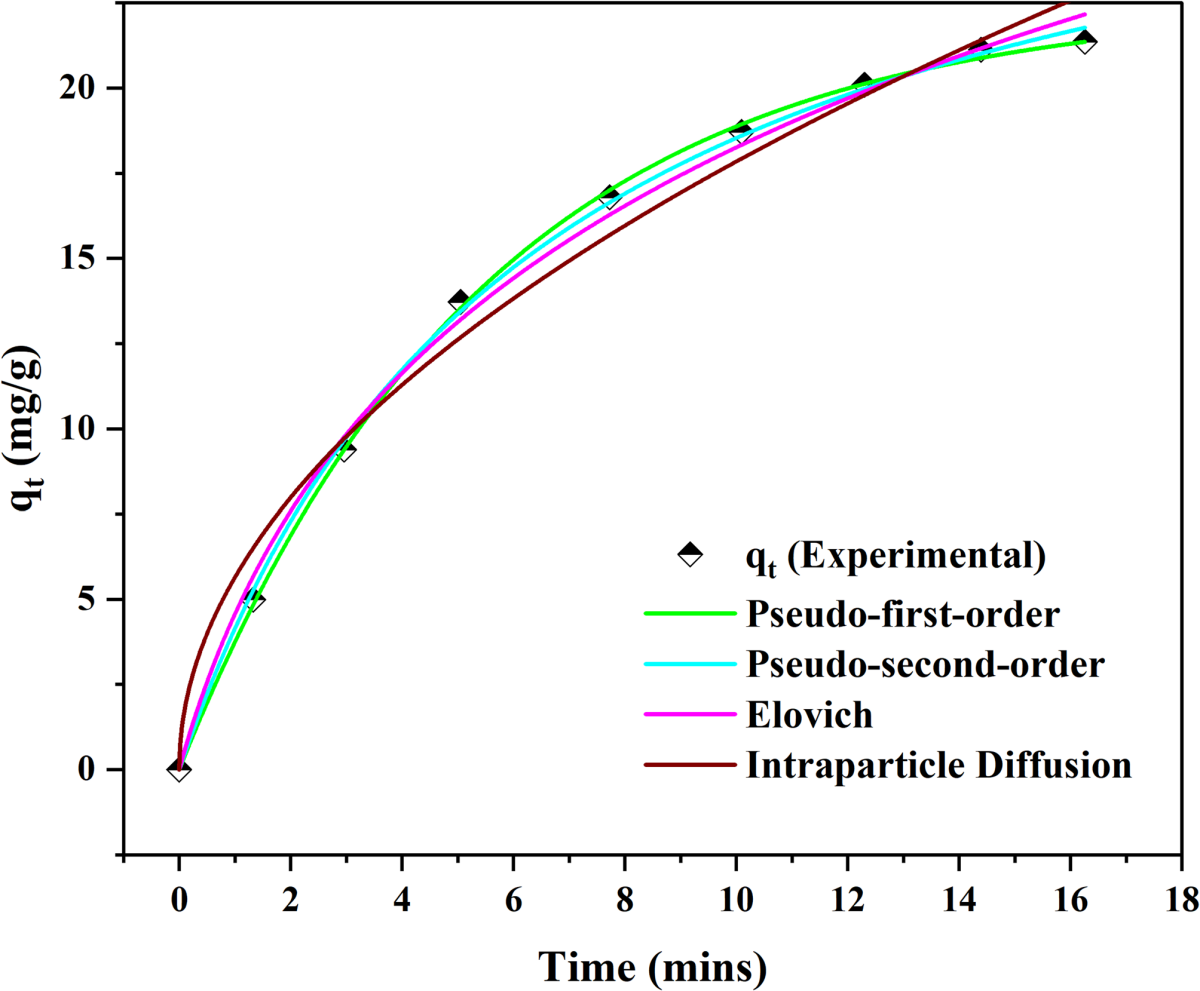
Non-linear kinetic models for ALZ adsorption process

The relationship between the adsorbent and adsorbate at designated time ranges was evaluated as demonstrated in Fig. 4.

The kinetic models are primarily employed to ascertain and explore the mechanisms involved in the adsorption process during ALZ adsorption. Table 5 shows the four kinetic models along with the parameters utilised for their evaluation.

**Table 5:** Constants of the kinetic models applied in ALZ removal

Entry	Model	Parameters	*R* ^2^
1	PFO	q_e_ = 22.5296 (mg/g) k_1_ = 0.1818 (min^− 1^) χ^2^ = 0.1178 SSE = 0.3203 ARE = 3.302	0.9996
2	PSO	q_e_ = 30.2001 (mg/g) k_2_ = 0.00526 (g/mg.min) χ^2^ = 0.0623 SSE = 0.1627 ARE = 2.370	0.9990
3	Elovich	a_E_ = 5.9459 mg/(g min) b_E_ = 1.1112 mg/(g min) χ^2^ = 0.0368 SSE = 0.4046 ARE = 4.025	0.9958
4	Intraparticle Diffusion	K_diff_ = 5.6417 (mg/g.min^0.5^) C = 0.6846 (mg/g) χ^2^ = 1.5630 SSE = 5.0075 ARE = 2.215	0.9842

The data presented in Table 5 indicates that the Elovich, PSO, intraparticle diffusion, and PFO models effectively characterise the removal of ALZ, as evidenced by the high R^2^ values. The intraparticle diffusion model demonstrated a high R^2^ value of 0.9842, with K_diff_ measured at 5.6417 (mg/g.min^0.5^) and C at 0.6846 (mg/g). These results suggest a rapid intraparticle diffusion process accompanied by minimal mass transfer resistance. The Elovich model, with an R^2^ = 0.9958, describes the process of ALZ removal to occur on energetically stable surfaces. Finally, the PFO with an R^2^ = 0.9996, along with the PSO with an R^2^ = 0.9990, indicates that the adsorption of ALZ was likely due to physisorption and chemisorption. The error analysis of the chi-square, square sum of errors and average relative were investigated to determine the model with the best quality, unexplained variability, and quantity of the size of errors [46]. From these error functions, it was determined that the PSO had the lowest error, and as such, the mechanism of adsorption was by chemisorption.

### Isotherm studies

**Fig. 5:**
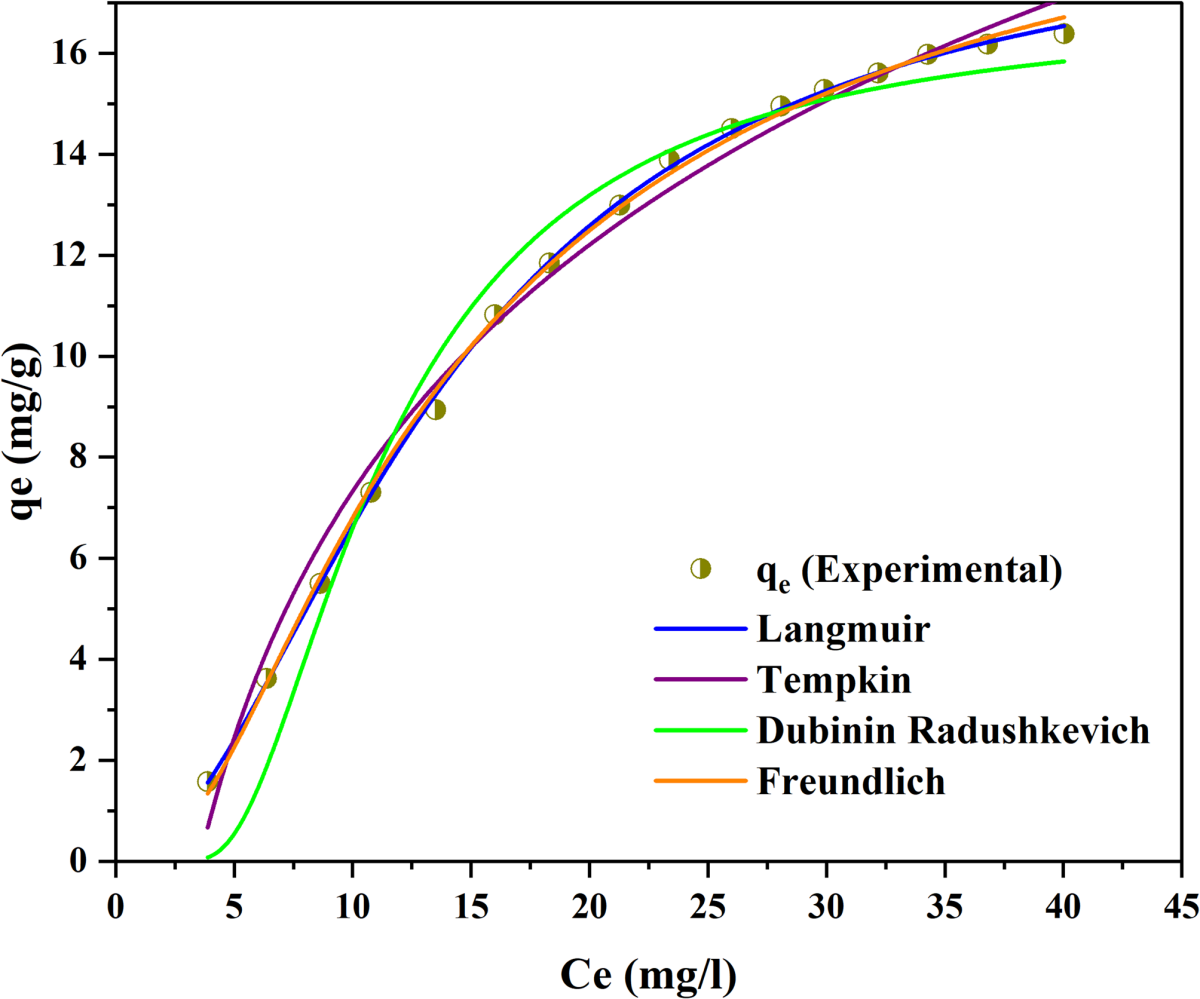
Non-linear isotherm models for ALZ adsorption process

To understand the mode of adsorption, non-linear isotherm models were investigated as shown in Fig. 5.

The plots of the non-linear isotherm models show a good correlation with the experimental values. The study was carried out on four models as elaborated in Table 6.

**Table 6:** Constants of the isotherm models applied in ALZ removal

Entry	Isotherm	Parameters	*R* ^2^
1	Freundlich	k_f_ = 20.8879 (mg/g) (L/mg)^1/n^, 1/*n* = 0.15053 χ^2^ = 6.3741 SSE = 0.00729 ARE = 0.01001	0.9985
2	Temkin	A = 0.2826 (L/g) B = 7.0466 (mg/l) χ^2^ = 18.7897 SSE = 0.00703 ARE = 0.04085	0.9880
3	Langmuir	k_L_ = 0.00685 (L/mg) q_max_ = 18.6747 (mg/g) χ^2^ = 4.2316 SSE = 0.1338 ARE = 0.12001	0.9995
4	Dubinin-Radushkevich	q_m_ = 16.8702 (mg/g) K_DR_ = 1.4281 (mol/kJ)^2^ E = 1.5175 (kJ/mol) χ^2^ = 26.4145 SSE = 0.01107 ARE = 0.1828	0.9735

The data presented in Table 6 indicates that all four isotherm models effectively characterise the adsorption of ALZ onto the activated carbon, as evidenced by the high correlation coefficient values (R^2^ >0.97). The 1/n value of 0.15053, being less than 1, indicates a strong affinity between adsorbate and adsorbent [47]. This suggests that adsorption was taking place on multilayer surfaces characterised by significant heterogeneity. The q_max_ = 18.6747 (mg/g) of the Langmuir, indicating monolayer coverage, also demonstrated that the adsorbent exhibited significant adsorption for the adsorbate. Furthermore, the Temkin model, which shows a reduction in adsorption heat resulting from the interactions between the adsorbate and adsorbent, demonstrated an (R^2^ = 0.9880). Lastly, the D-R exhibited an (R^2^ = 0.9735), which typically characterizes the mechanism of adsorption on heterogeneous surfaces with Gaussian energy distributions [48]. From the error analysis values of the chi-square, sum of square errors and average relative errors, it is observed that the best model is the Freundlich and thus the mode of adsorption occurs on multilayer surfaces.

### Central composite design

Various models and regression equations, including linear, two-factor (2 F), quadratic, and cubic models, were analysed to effectively predict the outcomes for ALZ removal. The alignment of each model was analysed, and the findings are presented in Table 7.

**Table 7:** Fit equations used in the study

Source	Sequential *p*-value	Lack of Fit *p*-value	Adjusted *R*²	Predicted *R*²	
Linear	< 0.0001	0.0030	0.8077	0.7450	
2FI	0.7383	0.0020	0.7864	0.7106	
Quadratic	< 0.0001	0.0522	0.9498	0.8613	Suggested
Cubic	0.6708	0.0134	0.9411	-0.6855	Aliased

The results presented in Table 7 indicate that the quadratic model is appropriate for predicting the removal of Alizarin, as it exhibits higher R^2^ and Adjusted R^2^ values compared to other models. The quadratic model is selected as the optimal model for this study. A lack of fit (0.0522) might be attributed to the model terms contributing well to the current data; however, they fail on new data.

### ANOVA for the quadratic model

The model factors were examined through ANOVA analysis. This analysis aimed to assess the interactive effects of four parameters on the removal of the dye. Confirmation was done by ANOVA, and this gave a choice of the quadratic model. The model was employed to determine the significance of the parameters and the differences in the statistics of the model. This study investigated the interaction of the process variables using ANOVA, as shown in Table 8.

**Table 8:** Interaction of the parameters based on statistics

Source	Sum of Squares	df	Mean Square	F-value	*p*-value	
Model	8519.12	14	608.51	40.17	< 0.0001	Significant
A-Time	77.83	1	77.83	5.14	0.0386	
B-pH	196.54	1	196.54	12.97	0.0026	
C-Dosage	5861.25	1	5861.25	386.91	< 0.0001	
D-Concentration	1160.65	1	1160.65	76.62	< 0.0001	
AB	95.65	1	95.65	6.31	0.0239	
AC	33.99	1	33.99	2.24	0.1549	
AD	3.63	1	3.63	0.2396	0.6316	
BC	5.09	1	5.09	0.3357	0.5709	
BD	4.28	1	4.28	0.2829	0.6026	
CD	83.36	1	83.36	5.50	0.0332	
A²	2.18	1	2.18	0.1441	0.7096	
B²	150.56	1	150.56	9.94	0.0066	
C²	730.54	1	730.54	48.22	< 0.0001	
D²	6.31	1	6.31	0.4164	0.5285	
Residual	227.24	15	15.15			
Lack of Fit	205.11	10	20.51	4.64	0.0522	Not significant
Pure Error	22.12	5	4.42			
Cor Total	8746.35	29				

The quadratic model’s fit quality for estimating dye removal as the output parameter was analyzed using statistical evidence (Table 8), which included the Lack of Fit assessment, probability value (P-value), and Fisher variation ratio (F-value). The mean squares (MS), degrees of freedom (DF), and sum of squares (SS) were also calculated. An F-value of 40.17 shows the statistical significance of the model. The probability of a “Model F-value” arising from noise is merely 0.01%. In addition, “Probability > F” less than 0.0500 show the statistical significance of the model. The results indicated that the terms C and D are significant, whereas A, B, AB, AD, AC, BD, BC, A^2^, B^2^, C^2^, and D^2^ are not significant. Additionally, values exceeding 0.1000 indicate the lack of significance of the model terms. Model reduction may enhance the model if various insignificant terms are present. An F-value of 4.64 indicates a 5.22% probability that such a large value could arise from random variation. The mathematical expression for the removal percentage is shown in Eq. 3.

3 $$ \begin{gathered} {\text{Removal }}\left( \% \right){\text{ }} = ~\,{\text{79}}.{\text{6833 }} + {\text{ 1}}.{\text{8}}00{\text{83 }}*{\text{ A }} + {\text{ }} - \hfill \\ {\text{2}}.{\text{86167 }}*{\text{ B }} + {\text{ 15}}.{\text{6275 }}*{\text{ C }} + {\text{ }} - \hfill \\ {\text{6}}.{\text{95417}}*{\text{D}} + {\text{ }} - {\text{2}}.{\text{445 }}*{\text{ AB }} + {\text{ }} - \hfill \\ {\text{1}}.{\text{4575 }}*{\text{ AC }} + {\text{ }} - 0.{\text{47625 }}*{\text{ AD }} + {\text{ }} - \hfill \\ 0.{\text{56375 }}*{\text{ BC }} + {\text{ }}0.{\text{5175 }}*{\text{ BD }} + {\text{ }} \hfill \\ {\text{2}}.{\text{2825 }}*{\text{ CD }} + {\text{ }} - 0.{\text{282}}0{\text{83 }}*{\text{ A}}^{{\text{2}}} + \hfill \\ {\text{ 2}}.{\text{34292 }}*{\text{ B}}^{{\text{2}}} + {\text{ }} - {\text{5}}.{\text{16}}0{\text{83 }}*{\text{ C}}^{{\text{2}}} + {\text{ }} - \hfill \\ 0.{\text{479583 }}*{\text{ D}}^{{\text{2}}} \hfill \\ \end{gathered} $$

### Model validation

The analysis of variance was used to obtain statistical factors that included standard deviation (Std. Dev), mean, and coefficient of variation (C.V) as shown in Table 9.

**Table 9:** Model validation based on analysis of variance

*R* ^2^	*R*² (Adjusted)	*R*² (Predicted)	Adequate Precision	Std. Dev.	Mean	C.V. (%)	Press
0.9740	0.9498	0.8613	27.5510	3.89	76.82	5.07	1213.31

Table 9 shows a coefficient of variation of 5.07%, which shows the evaluation precision, credibility and estimation standard error of the experimental data. It is also utilised to assess the accuracy of the model. Std. Dev between the modeled and actual results is 3.89%. PRESS represents the proportion of the model at each point, with lower values indicating better performance. An R^2^ = 0.9740 indicates a stable fit of the experimental data for Alizarin removal. The R^2^ (Predicted) = 0.8613 does not align closely with the R^2^ (Adjusted) = 0.9498, which is typically anticipated. This may suggest a potential issue with the data or current model. Considerations must be made for outliers, response transformation and model reduction. Adequate Precision quantifies the signal-to-noise ratio, determined by dividing the range of predicted values (the difference between the maximum and minimum predicted responses) at the design points by the average prediction error [49]. The precision of Alizarin removal is 27.5510, indicating a sufficient signal. A ratio exceeding 4 is favourable, suggesting that the model’s mean is capable of delivering adequate performance in line with the predictions [50]. The findings indicated that this model is applicable for navigating the design space.

### Interaction study between the variables

**Fig. 6:**
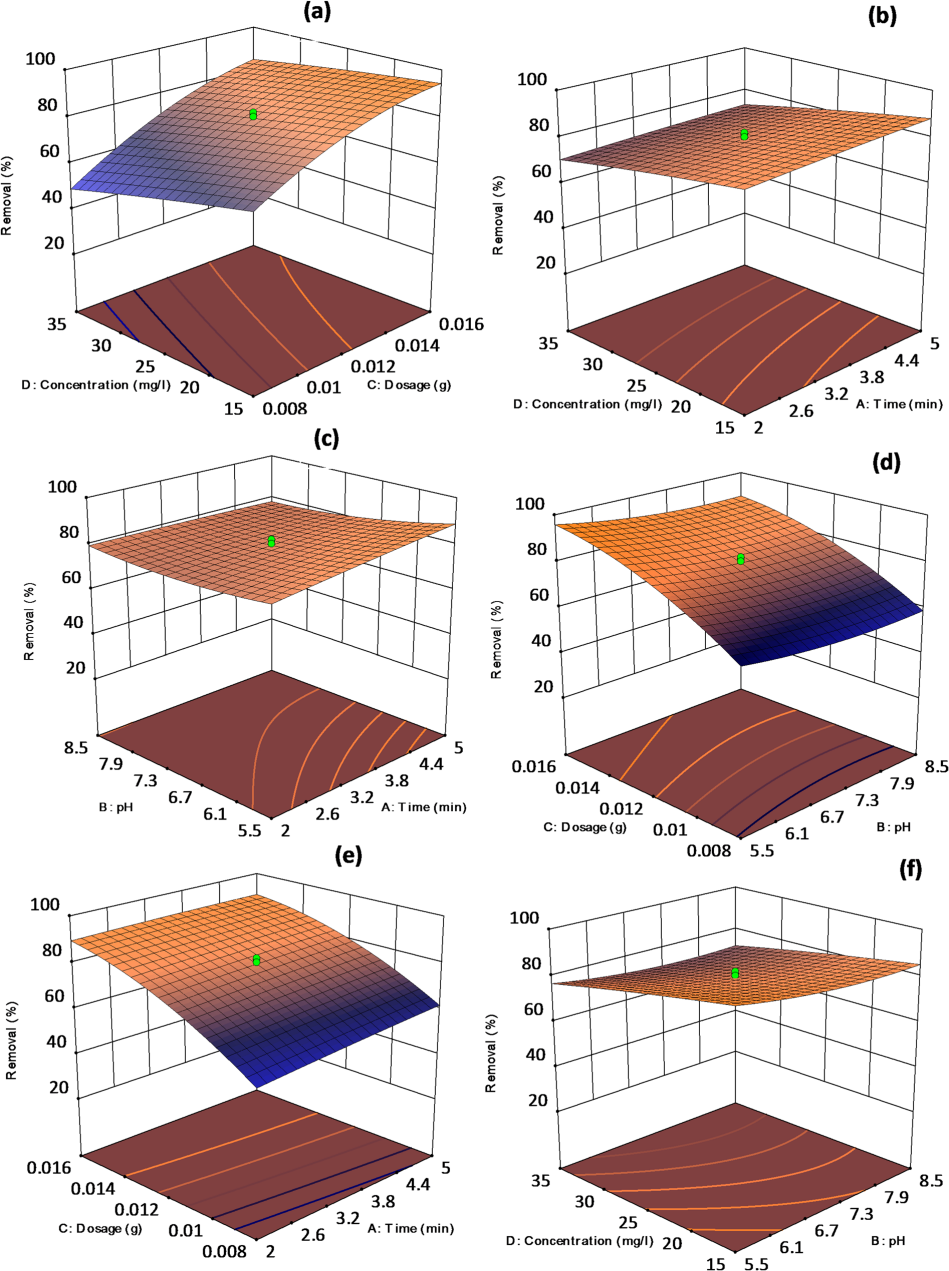
Interaction of the process variables used in ALZ removal based on 3D surface plots

This study presents 2D contour plots and 3D surface response plots as graphical expressions of the regression equation. These visual tools are employed to identify optimal amounts of various factors and to enhance understanding of the interactions among different parameters affecting the response. The results of the interactions among the four factors are illustrated graphically as shown in Fig. 6.

Figure 6a illustrates the impact of the interaction between dosage and concentration on the removal of ALZ. The removal rate exhibited a positive correlation with both concentration and dosage increments. Figure 6b shows the interaction between concentration and time, and it is observed that the removal increases as the time of contact increases, as the dye interacts strongly with the adsorbent, thus leading to a high removal percentage. Figure 6c shows the effects of pH and time, and it is observed that the removal increases with an increase in pH. Figure 6d illustrates that the removal rate decreased with a reduction in adsorbent dosage and increased with a rise in pH. Figure 6e illustrates the effects of dosage and time, and from the observations, it is seen that as the dosage and time increase, the removal percentage also increases. This is attributed to the increase in the binding sites of the adsorbent as the dosage increases and a higher time of contact. Figure 6f illustrates that the removal of the dye increases with decreasing dye concentration and pH value. Furthermore, the findings of this study align with previous research indicating that the removal of ALZ increased as pH decreased (under acidic conditions), leading to a reduction in dye pollutant concentration and an increase in adsorbent dosage.

**Fig. 7:**
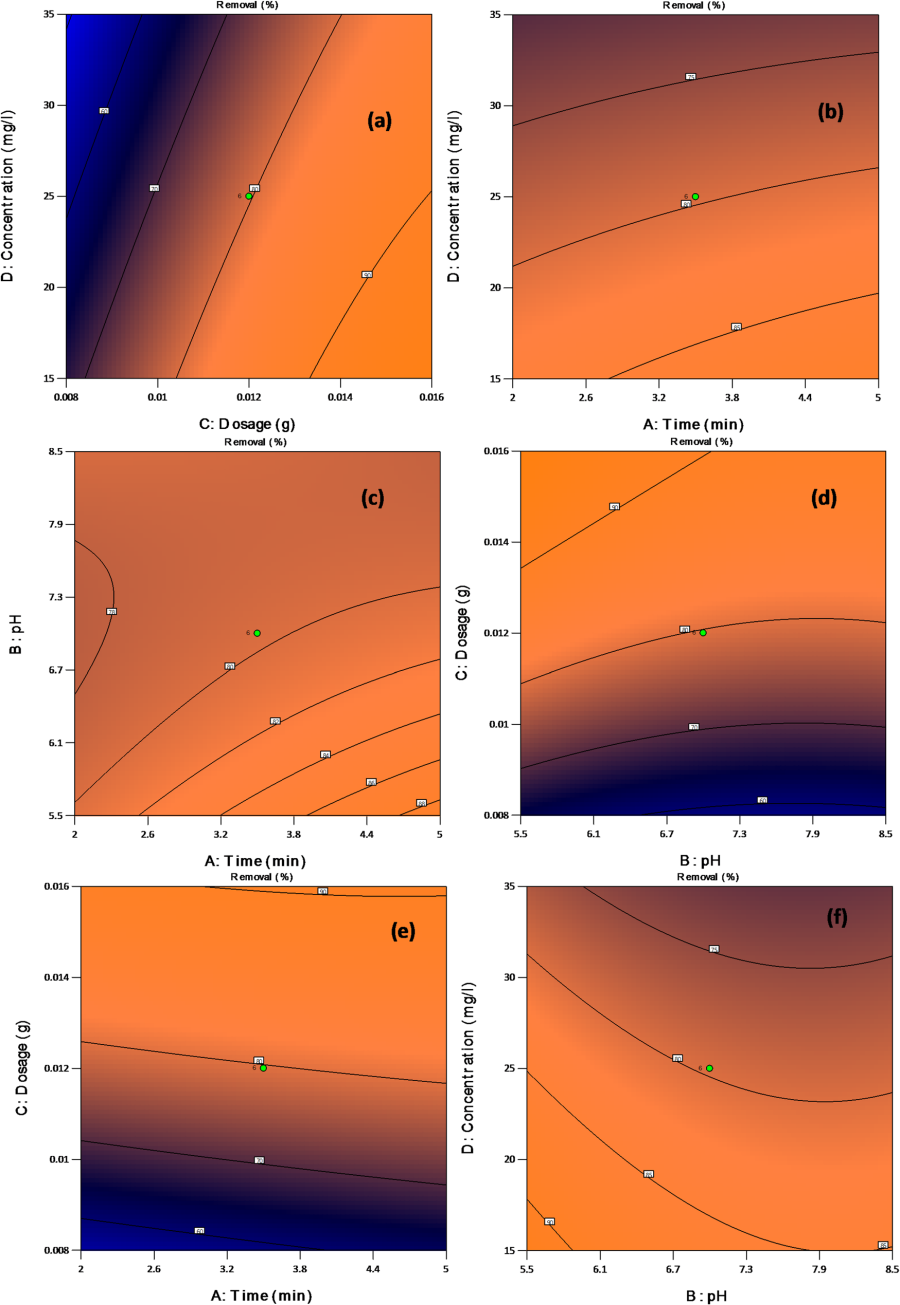
Interaction of the process variables used in ALZ removal based on 2D contour plots

Figure 7 presents the 2D contour plots illustrating the percentage removal of ALZ. These findings align closely with the 3D surface plots.

**Fig. 8:**
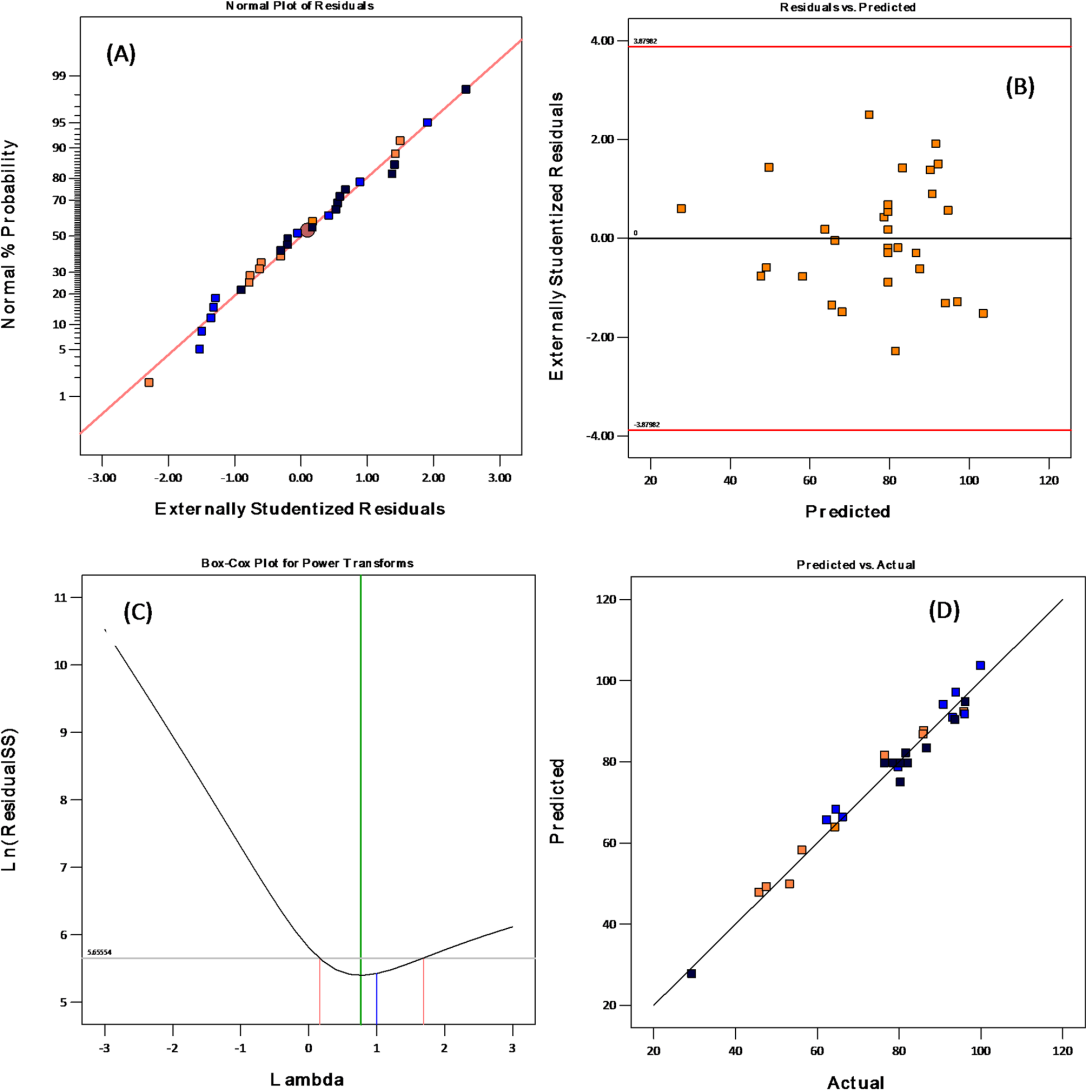
Plots of actual, predicted, residuals, and Box-Cox analysis

Figure 8 displays the plots for actual values, predicted values, normal plot of residuals, and Box-Cox transformation of the residuals related to the experimental data in the study of removal percentage.

Figure 8a illustrates a linear relationship in the data distribution, suggesting that the experimental results correspond with the predicted response values. Figure 8b further confirms that the distribution of studentized residuals in relation to predicted values does not display any discernible pattern. The obtained residuals demonstrate typical variability, thereby supporting the validity of the proposed model. Figure 8c illustrates the box–cox plot, and it indicates a lambda value near 1, implying that no transformation is required for determining the adsorption capacity. The data scattering illustrated in Fig. 8d demonstrates a linear relationship, suggesting that the experimental results align closely with the predicted data.

**Fig. 9:**
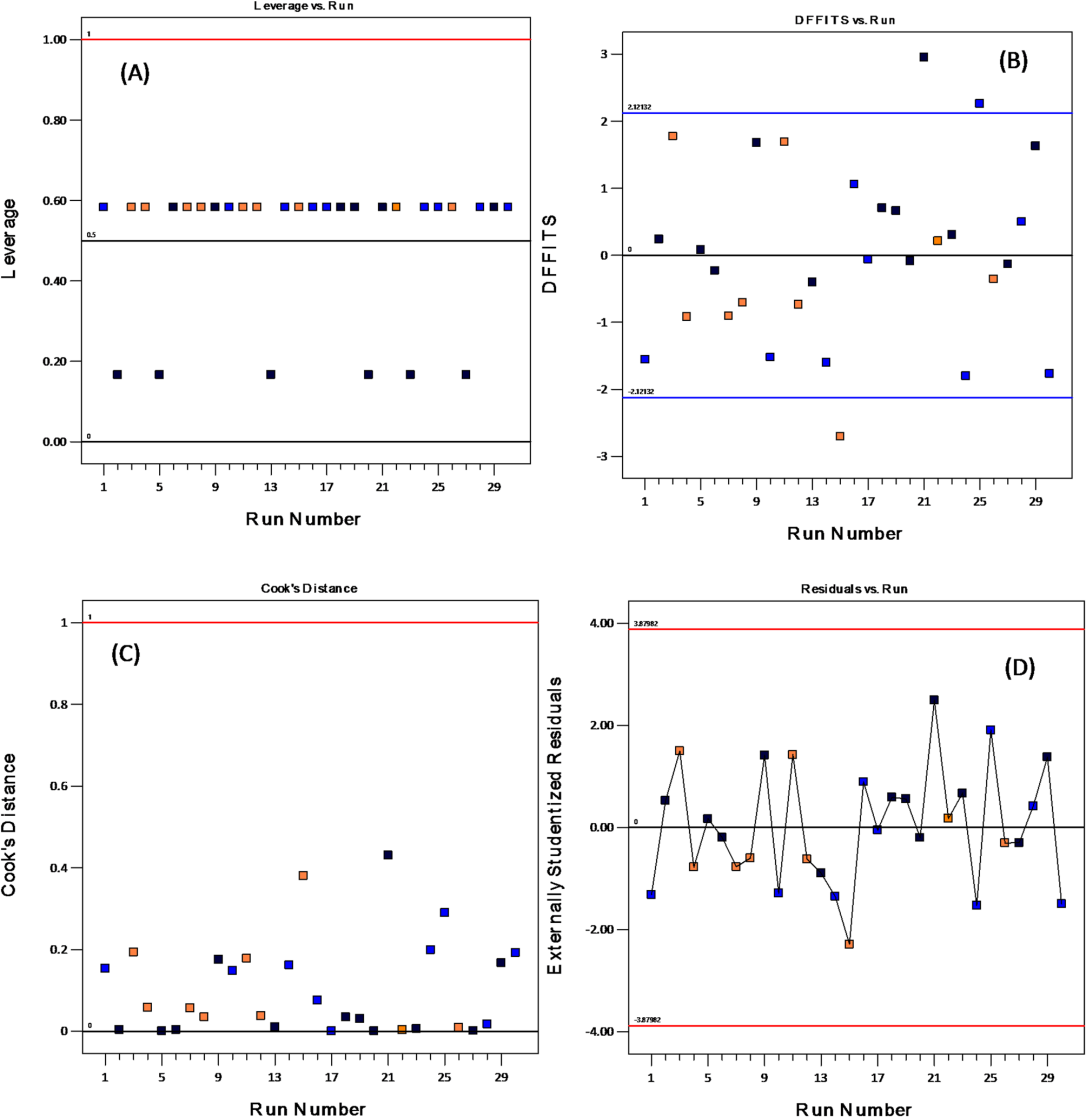
Plots of Cook’s distance, DFFITS, residuals and leverage against run number

Figure 9 illustrates the plot of studentized residuals, Cook’s distance against the run number, leverage versus run, and DFFITS versus run.

The analysis of leverage versus runs indicates that no data points exceed the average leverage, as such points would affect at least one model parameter. The DFFFITS algorithm quantifies the influence of each observation on the expected value. Cook posits that it can be translated to a distance. DFFITS can take on both positive and negative values, unlike Cook’s distances. When the value equals zero, the specified point lies exactly on the regression line. The use of leverage facilitates this outcome. The mathematical difference exists between the expected value derived from observations and the forecast value absent from observations. The graphs illustrate that DFFITS values (Fig. 9b) fell within the size-adjusted acceptable range, except for two values observed at runs 21 and 25. Nevertheless, these values were only marginally above the acceptable range and do not significantly impact the overall reliability of the model. The cooks plot against the run indicates that most data points fell below 0.2, suggesting that the model adequately describes the removal percentage (Fig. 9c). Furthermore, the plots of residuals versus run number and residuals versus predicted values for removal percentage are presented in Fig. 9d.

**Fig. 10:**
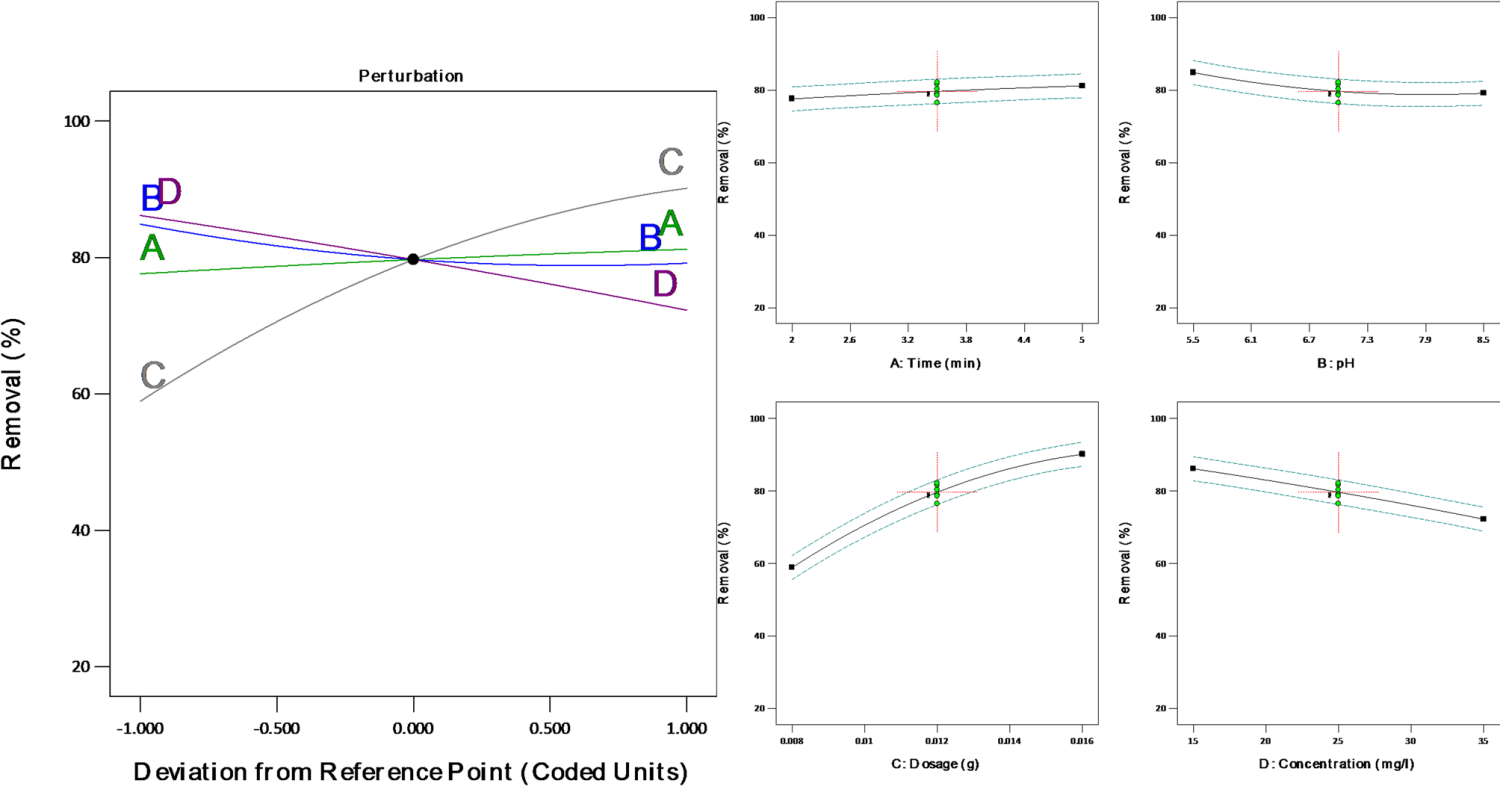
Variation of the process variables based on the perturbation plot

The perturbation plot and interactive effects of the individual parameters on the removal of dye are shown in Fig. 10.

The slope of each curve indicates the sensitivity of the responses to the respective factors. The perturbation plot describes the interaction of the four input parameters in the removal efficiency of ALZ.The perturbation plot highlights that the four input variables are in agreement with the removal efficiency of ALZ by the banana peel activated carbon. The plot shows the removal of ALZ to increase as the pH tends to be acidic, which is mainly attributed to the strong surface charge which arises due to the change in functional groups of the adsorbent. In addition, it is observed that at low adsorbate concentration the removal efficiency increases, this is because more ALZ interact with the surface of the adsorbent, hence great adsorption. It is worth noting that the increase in dosage had a significant effect on the removal of the ALZ due to the availability of more active sites. Lastly, removal efficiency was also increased as the contact time was varied since the adsorbate spends more time on the surface of the adsorbent, which leads to high adsorption.

### Desirability

**Fig. 11:**
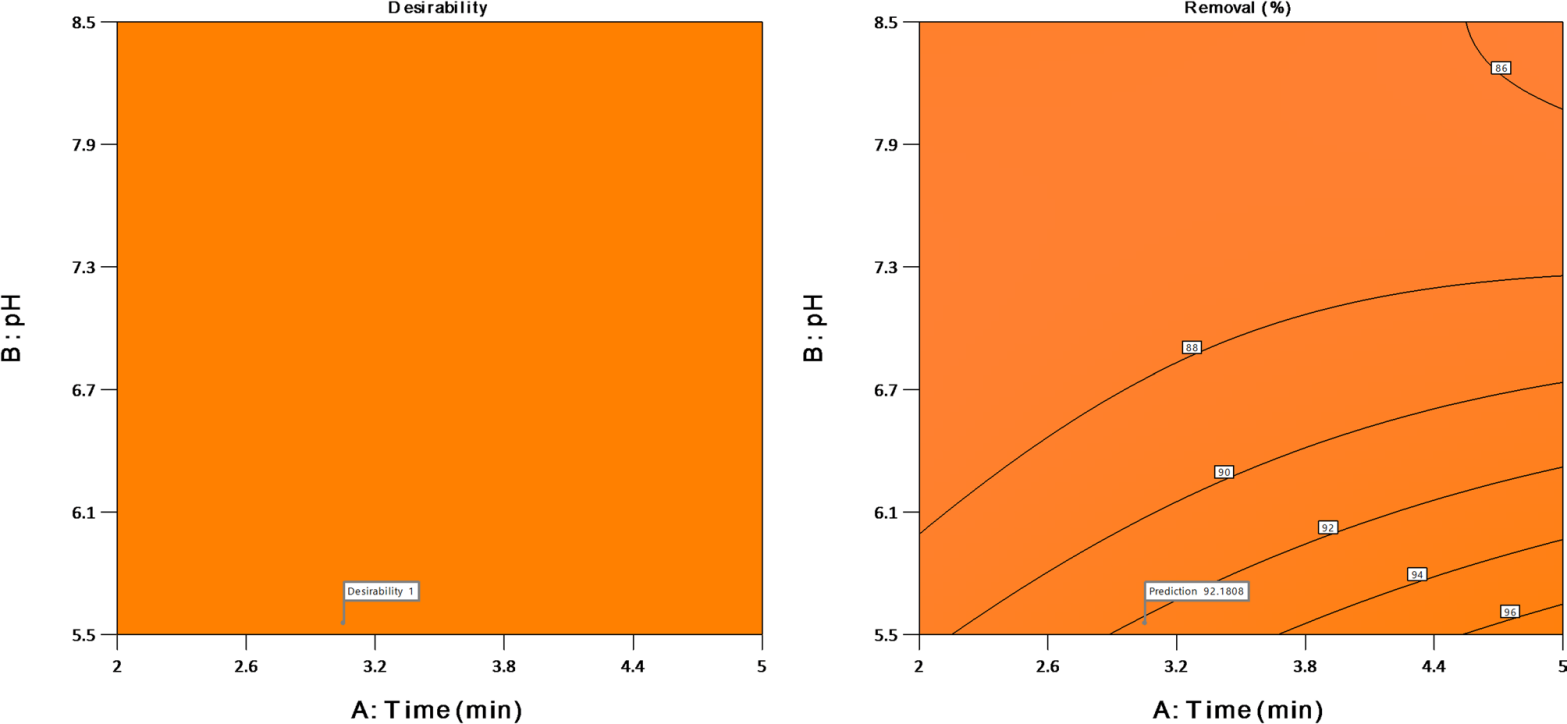
A representation of some of the optimal process variables

The optimal conditions for various factors, including pH, concentration, time, and dosage, were predicted for the removal of the dye were further investigated using the desirability function (DSF). The primary objective of the model was to achieve maximum percentage dye removal. Figure 11 displays the 3D and the 2D plots of desirability under optimal conditions.

The findings in Fig. 11 indicate that the interaction between ALZ concentration and pH exerts the most substantial influence on decomposition.

**Fig. 12:**
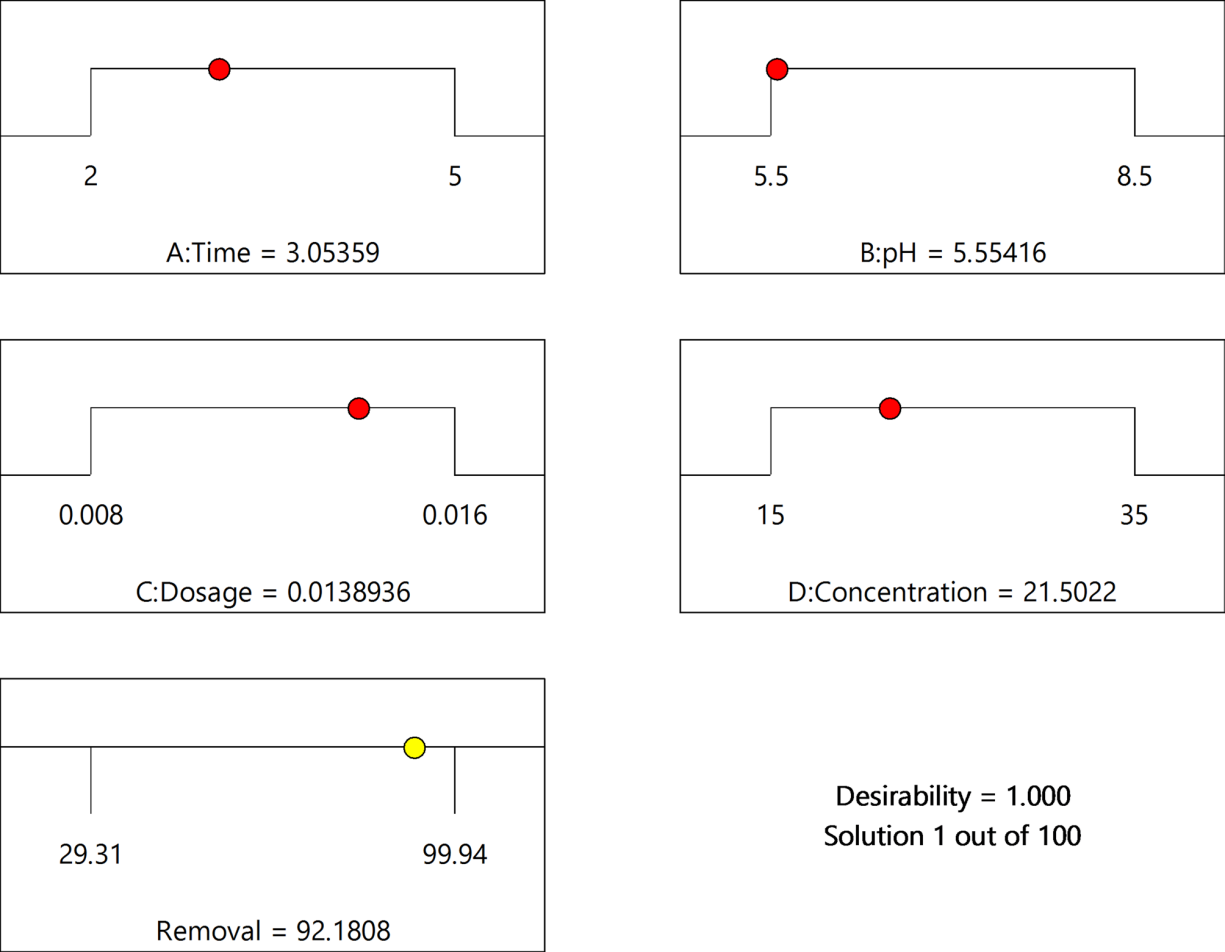
Desirability function for the optimal process variables

Figure 12 illustrates the optimal values for each parameter and the corresponding desirability function.

Figure 12 illustrates that the maximum removal percentage of Alizarin is 92.1808%, achieved with an overall desirability of 1 under the conditions of Time: 3.05359, pH: 5.55416, dosage: 0.00138936, and concentration: 21.5022 ppm. The DSF of 1 demonstrated suitable conditions for the removal of the dye by the adsorbent. The desirability of 1 validates the model as an effective technique for designing optimal conditions. The results correlated with the predicted outcomes, thereby confirming the validity and adequacy of the model. The optimal conditions for the removal of the dye are presented in Table 10. The optimal conditions for the highest removal percentage of Alizarin are a time of 3.054 min, a pH of 5.554, a concentration of 21.502 mg/l, and a dosage of 0.014 g.

**Table 10:** Best optimization process variables for ALZ removal

Number	Time	pH	Dosage	Concentration	Removal	Desirability	
1	3.054	5.554	0.014	21.502	92.181	1.000	Selected
2	2.000	8.500	0.008	15.000	65.608	1.000	
3	5.000	5.500	0.016	35.000	92.280	1.000	
4	3.500	7.000	0.012	25.000	79.683	1.000	
5	2.000	5.500	0.016	15.000	97.081	1.000	
6	5.000	8.500	0.008	15.000	68.187	1.000	
7	2.000	5.500	0.008	15.000	66.349	1.000	
8	5.000	5.500	0.016	15.000	99.401	1.000	
9	5.000	8.500	0.016	15.000	90.835	1.000	
10	5.000	5.500	0.008	35.000	58.247	1.000	

### Modeling of process parameters using artificial neural networks

**Fig. 13:**
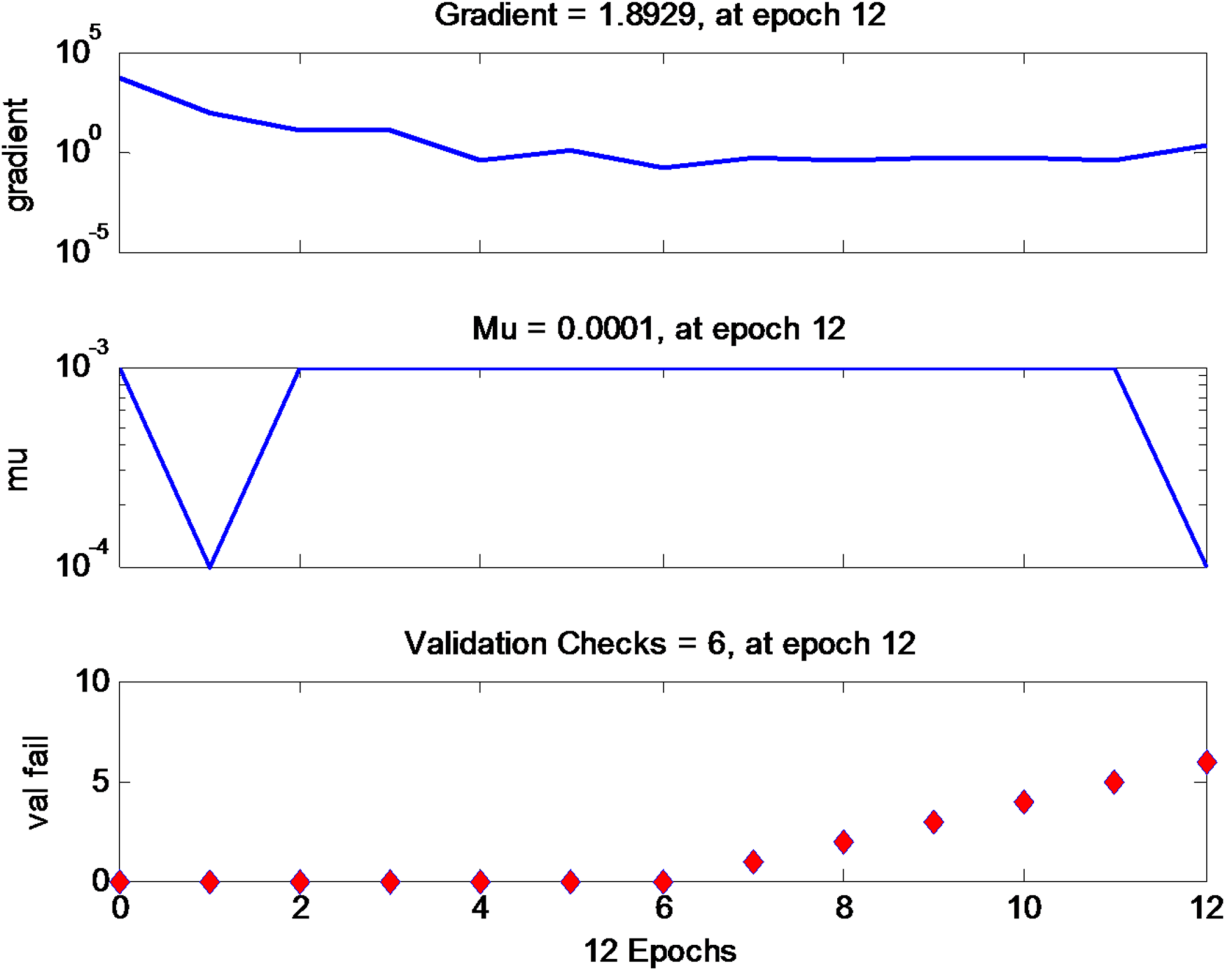
A validation plot of the artificial neural network

The development of the network with the ideal performance is obtained when the optimal number of neurons is selected. To enhance the model, the independent input neurons and the dependent output neuron remain fixed, while adjustments are made to the hidden layer neurons. The dataset is divided into 70% (training set), 15% (validation set), and 15% (testing set). The mean squared error displayed a significant distribution over the entire network, as shown in Fig. 13.

The plot illustrating the training sets in relation to failure is constructed utilising the gradient, validation, and mu, which are obtained from their correlation with the epochs as shown in Fig. 13. The gradient across the Epochs shows a consistent decrease with each passing Epoch, while the mu stays mostly stable, with a minor reduction noted after the 12th Epoch. The validation against the Epoch reveals a failure after the 12th Epoch, as shown by the rise beyond the optimal point.

**Fig. 14:**
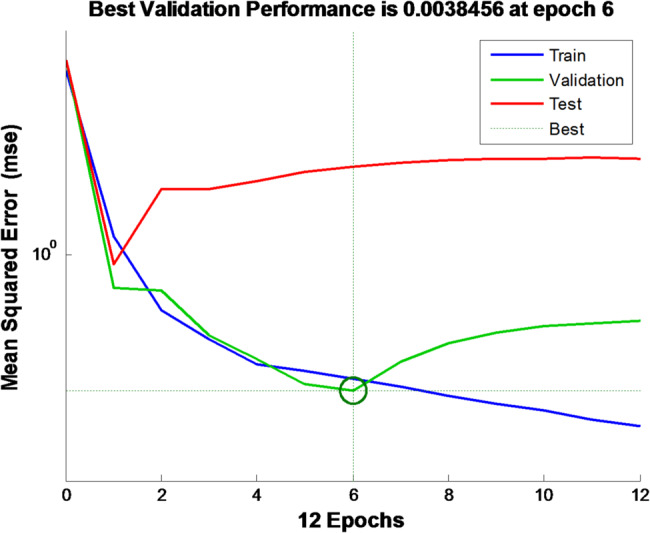
A representation of the ANN, mean squared error

The evaluation of the training set’s performance is conducted through a graphical representation of MSE in relation to the epoch number under ideal conditions, offering valuable insights into the model’s validation process as shown in Fig. 14.

The data from Fig. 14 suggests that the best performance validation, as indicated by the corresponding MSE, is approximately 0.0038456 during the 6th epoch iteration. A minor mean-squared error analysis suggested that the training network did not experience any overfitting problems. An elevation in the test curve before the initial ascent of the validation curve suggests the presence of overfitting.

**Fig. 15:**
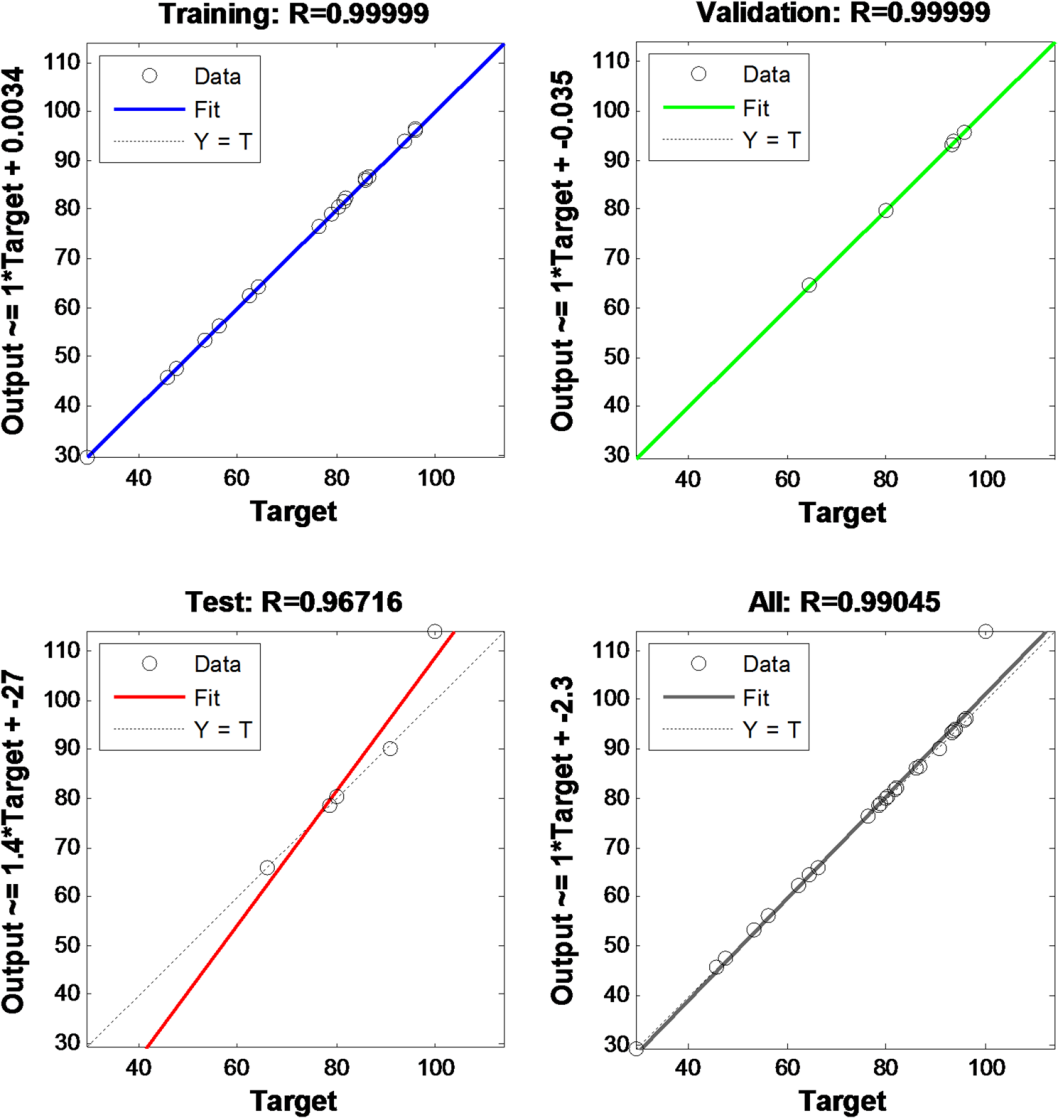
ANN plots based on experimental data training

Figure 15 illustrates the correlation coefficient of the model based on Training (R^2^ = 0.99999), Validation (R^2^ = 0.99999), Test (R^2^ = 0.96716) and All (R^2^ = 0.99045).

Figure 15 presents the ANN regression graphs that depict the training, validation, test, and overall data sets in a normalised format. Data normalisation was conducted at the outset. The observed correlations were as follows: training (0.99999), testing (0.96716), validation (0.999999), and total (0.99045). The correlation coefficients neared one across all datasets, indicating a satisfactory fit for each dataset. As a result, the output of the network accurately represented the removal percentage. It is worth noting that the model experienced over-training, and as such, early stopping plus data splitting was done during the optimization process.

### Comparison of the predictive models

The predictive models were compared based on their values against the actual value, as shown in Table 11.

**Table 11:** Comparative analysis of actual versus predicted values for the removal of Alizarin

Run	Time (mins)	pH	Dosage (g)	Concentration (mg/l)	Actual value	RSM Pred	ANN Pred
1	2	8.5	0.016	15	90.86	94.09	89.95
2	3.5	7	0.012	25	81.62	79.68	81.60
3	5	5.5	0.016	35	95.9	92.28	95.78
4	5	5.5	0.008	35	56.27	58.25	56.27
5	3.5	7	0.012	25	80.32	79.68	80.34
6	6.5	7	0.012	25	81.65	82.16	81.63
7	2	5.5	0.008	35	45.84	47.79	45.84
8	2	8.5	0.008	35	47.59	49.12	47.59
9	3.5	10	0.012	25	86.78	83.33	86.51
10	2	5.5	0.016	15	93.92	97.08	93.89
11	5	8.5	0.008	35	53.27	49.8	53.27
12	2	5.5	0.016	35	86.06	87.66	86.18
13	3.5	7	0.012	25	76.48	79.68	76.49
14	2	8.5	0.008	15	62.3	65.61	62.30
15	5	8.5	0.016	35	76.5	81.57	76.50
16	5	8.5	0.016	15	93.1	90.83	93.12
17	2	5.5	0.008	15	66.22	66.35	66.02
18	3.5	7	0.004	25	29.31	27.79	29.31
19	3.5	4	0.012	25	96.22	94.78	96.26
20	3.5	7	0.012	25	78.96	79.68	78.94
21	0.5	7	0.012	25	80.35	74.95	80.37
22	3.5	7	0.012	45	64.32	63.86	64.32
23	3.5	7	0.012	25	82.13	79.68	82.16
24	5	5.5	0.016	15	99.94	99.60	99.40
25	3.5	7	0.012	5	96.1	91.67	96.07
26	2	8.5	0.016	35	85.95	86.73	86.12
27	3.5	7	0.012	25	78.59	79.68	78.52
28	5	5.5	0.008	15	79.8	78.71	79.83
29	3.5	7	0.02	25	93.66	90.3	93.66
30	5	8.5	0.008	15	64.58	68.19	64.52

Table 11 shows that both the central composite design and artificial neural networks predicted output values are very close to the actual output values.

**Fig. 16:**
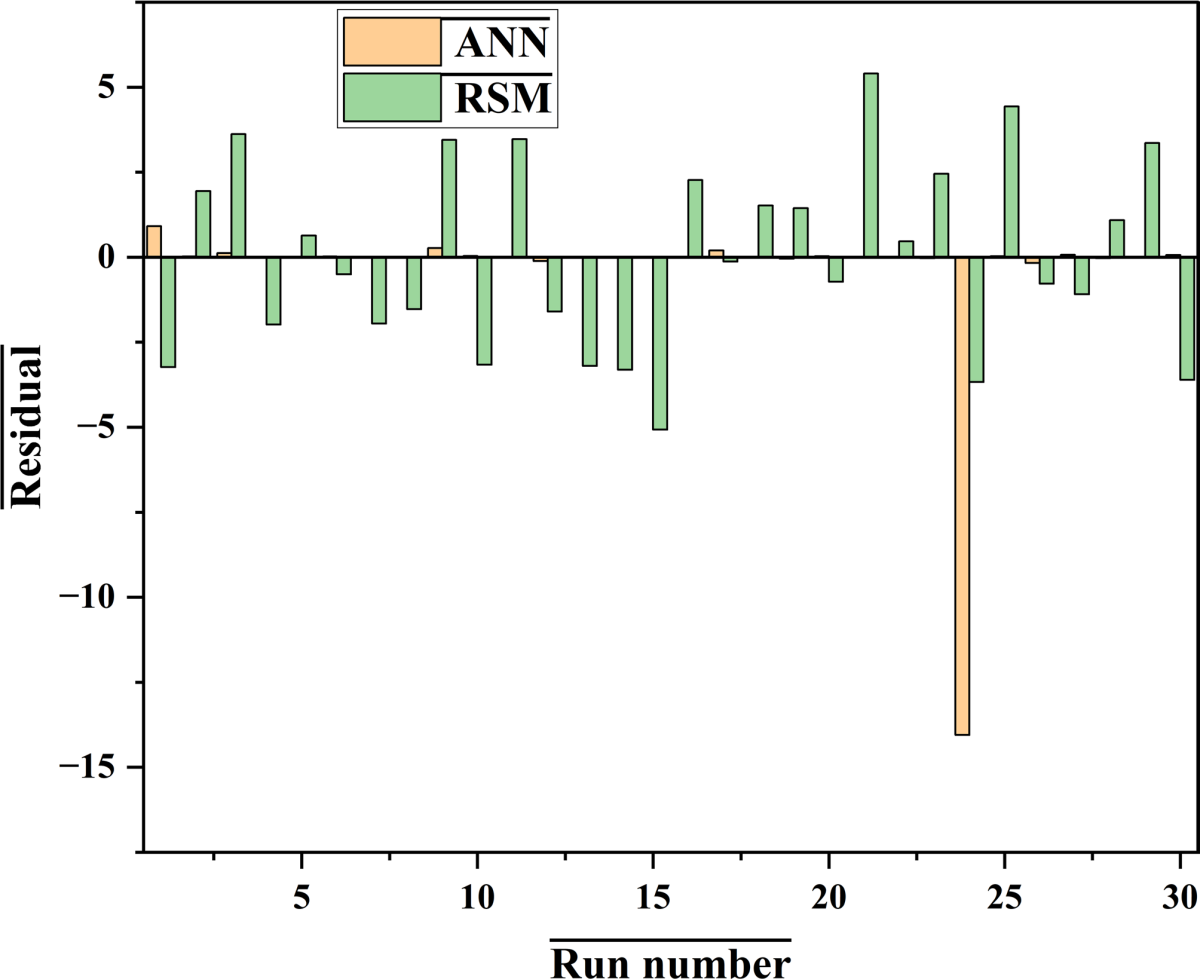
Differences in residual errors of CCD and ANN models

Figure 16 describes the difference in the ANN and CCD residual errors over a range of the experiment run numbers.

**Fig. 17:**
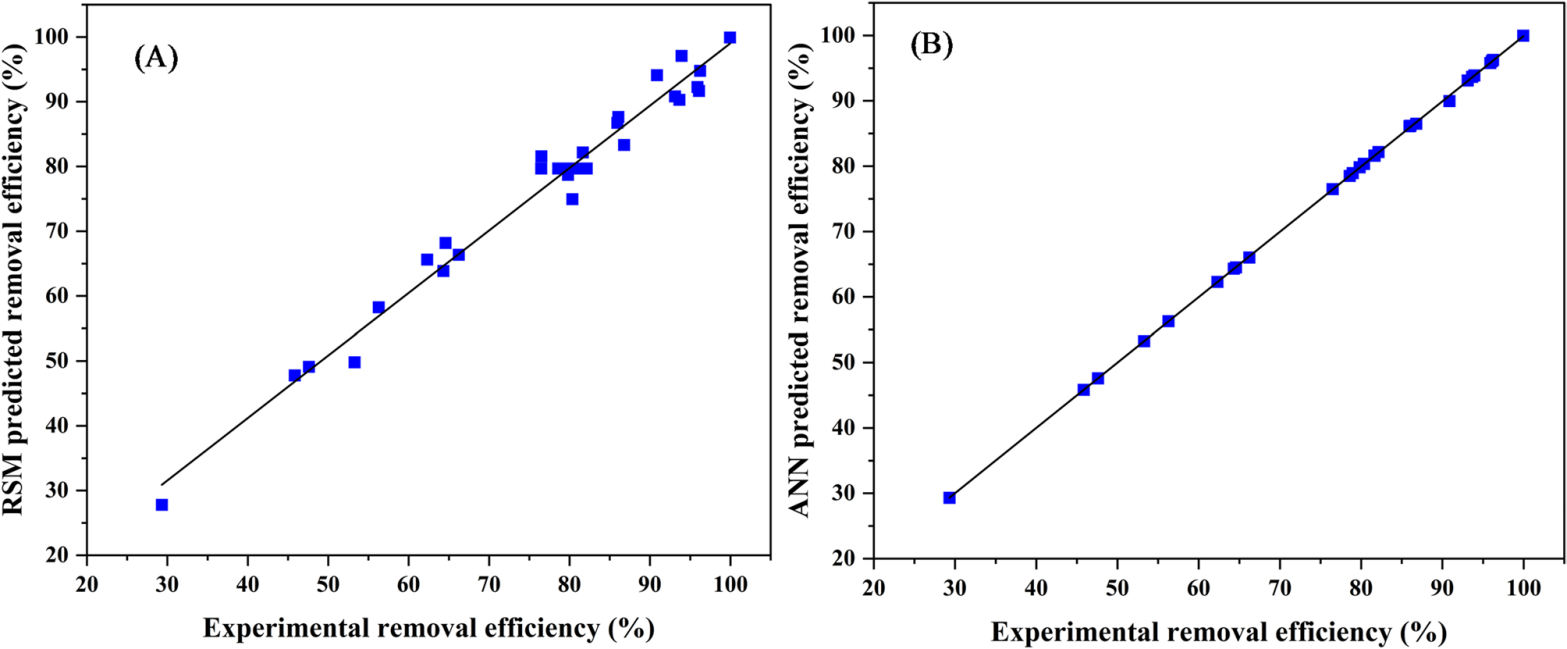
A regression plot of predicted against experimental data

The residual error plot shows in Fig. 16 that the ANN had values that were close to zero, hence it had a better performance compared to the CCD. This can either be based on the fact that the ANN have validation, training, and testing data sets, which enable it to learn more and adapt to the data given. It is worth noting that from Fig. 16, very few residual points lie far away from the middle line, thus showing ANN superiority. The comparative data, which was used to determine a correlation between the predicted and experimental values, was further illustrated graphically as shown in Fig. 17A and B.

The plot obtained from Fig. 17 (A and B) shows a strong relationship between the predicted and experimental data for both RSM and ANN. The axial and factorial points showed no deviations from the diagonal. The study further carried out statistical analysis using error functions to compare the model compatibility with the experimental data.

The analysis was carried out using seven error functions to assess the model’s precision abilities, as shown in Table 12. The models that were investigated include R^2^, Adj. R^2^, Pearson’s r, SSE, MSE, RMSE, and MAE.

**Table 12:** Comparison of RSM and ANN using error functions

Entry	Error functions	RSM	ANN
1	R^2^	0.97574	0.99989
2	Adj.R^2^	0.97488	0.99989
3	Pearson’s r	0.9878	0.99995
4	SSE	202.1435	0.93923
5	MSE	0.0003162	0.000002229
6	RMSE	0.01778	0.001513
7	MAE	0.003267	0.001493

Initially, these error functions are analyzed based on the lower the values, which show the model’s predictive efficiency. It is observed from Table 12 that the two models showed small deviations, which were indicative of low errors. R^2^ are not supposed to be lower than 0.8 for comparison of experimental and predicted values, since this compromises the model’s reliability. Similarly, the Adj.R^2^ was determined to assess the overestimation of R^2^, which, based on the obtained data, was high, thus showing the model’s importance. It is worth mentioning that the higher R^2^ and Adj.R^2^ values, the better the predictions obtained from a given model. From the statistical comparison of the two models, it is concluded that the ANN is slightly superior to RSM.

### Mechanism of adsorption

**Fig. 18:**
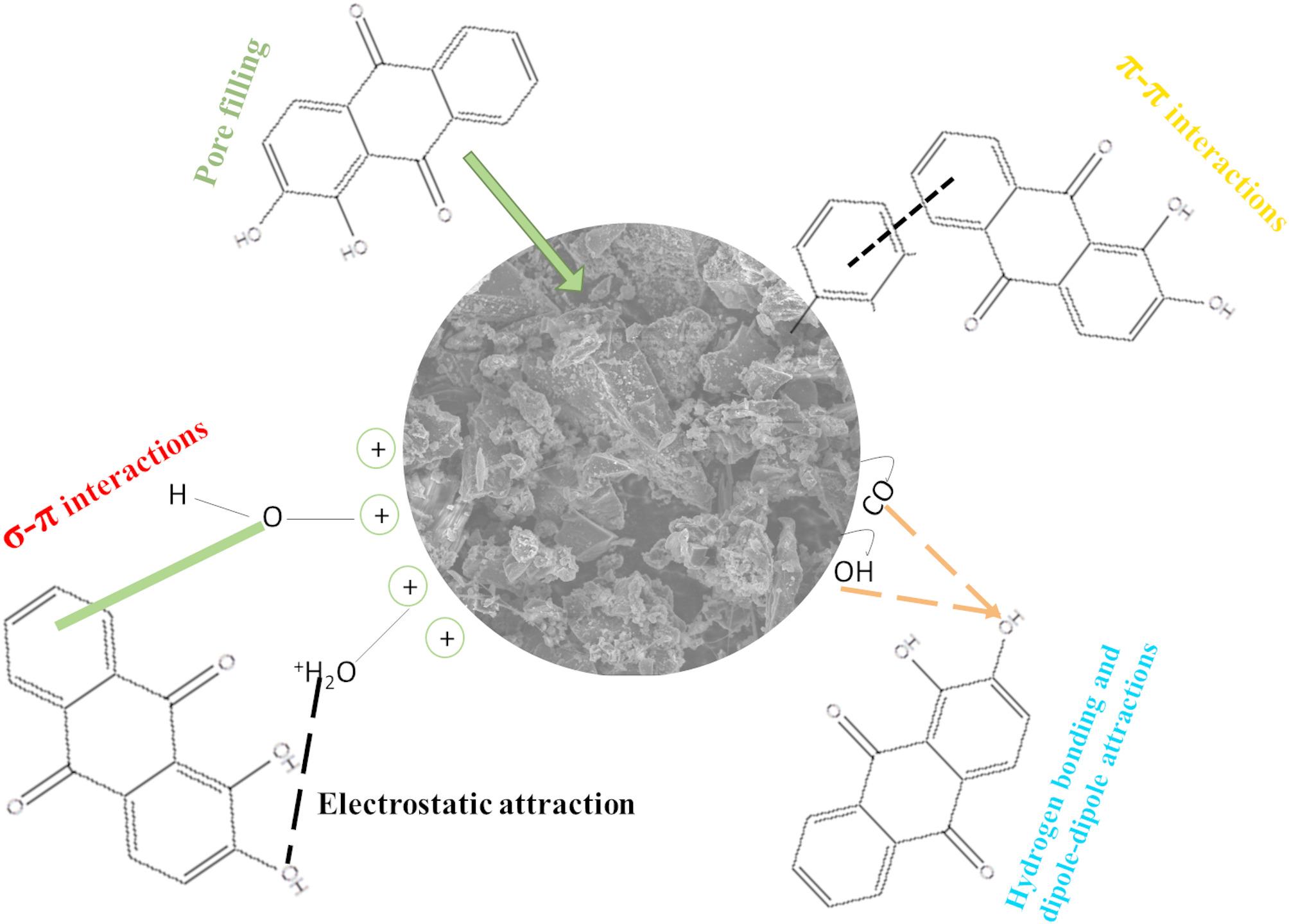
Possible adsorption mechanisms in the removal of ALZ

The mechanism of adsorption can generally be elaborated using the data obtained from FTIR, BET, XRD, and SEM analysis. These techniques highlight the possibility of hydrogen bonding, pore filling, electrostatic attraction, dipole-dipole interactions, π-π, and σ-π interactions as illustrated in Fig. 18.

FTIR spectra showed the presence of the hydroxyl, carbonyl and aromatic carbons, which are important in the creation of π-π and σ-π interactions, which highlight the involvement of oxygen-containing functional groups. BET analysis through N_2_ physisorption isotherm showed that the adsorbent had a large surface area and pore sizes where the dye could easily interact with the adsorbent through pore filling and electrostatic attraction, which was a result of enough active sites. It is also backed by the Dubinin Radushkevich isotherm, which demonstrates adsorption through pore filling. Furthermore, the SEM image illustrates a porous, rough structure of the adsorbent, which shows that some adsorbate molecules are intercepted on the surface while others are trapped in the pores, which also shows pore filling, π-π, and σ-π interactions. Lastly, using XRD analysis, hydrogen bonding through the presence of amorphous, non-crystalline structure with the presence of graphitic materials gives rise to the hydroxyl functional group, which creates a bond, thus giving rise to hydrogen bonding and dipole-dipole interactions.

### Application of banana peel-activated carbon on real water samples

**Fig. 19:**
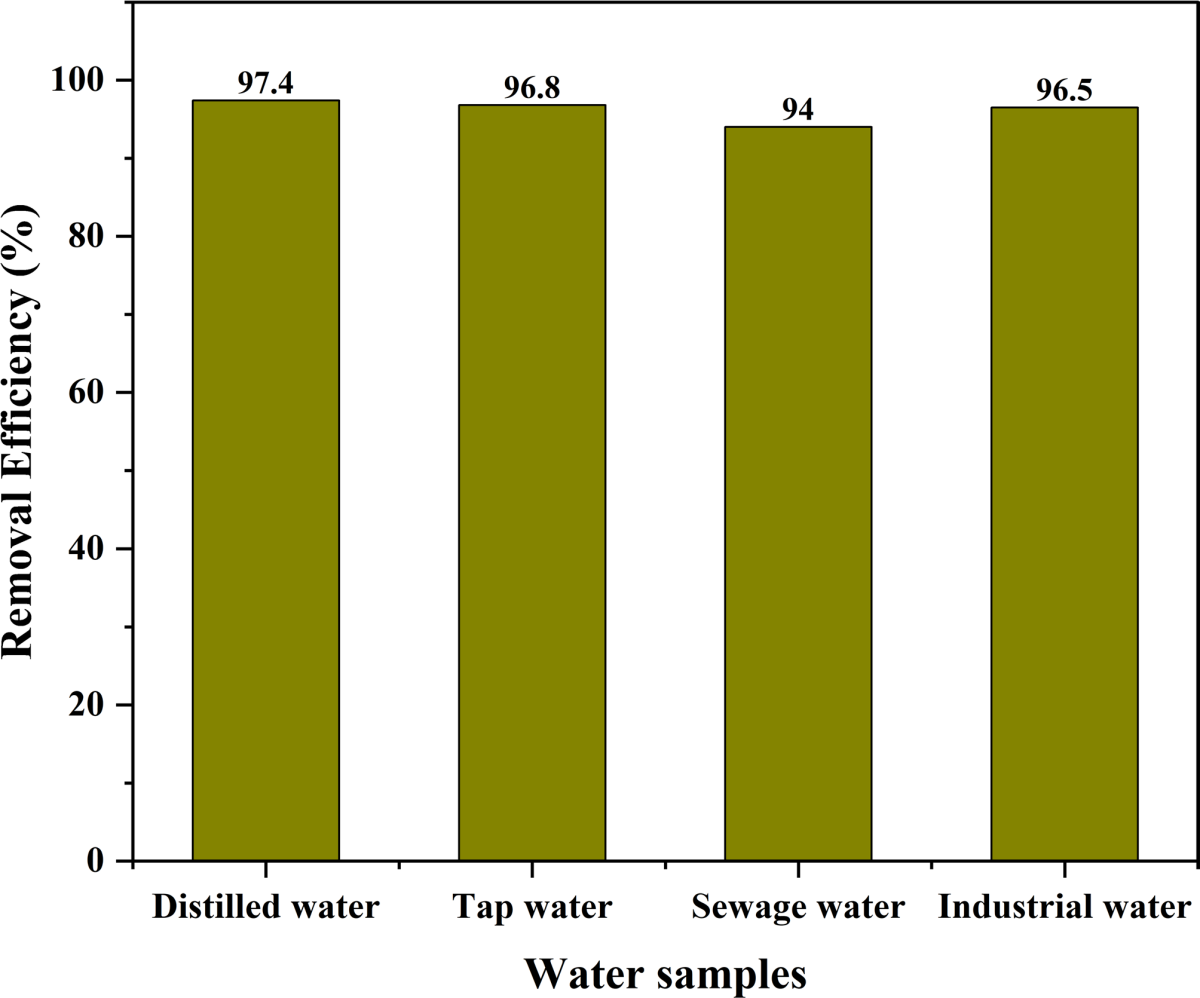
Removal of alizarin from real water samples

Four water samples were collected from areas around Kampala, Uganda, to investigate the practical applicability of the adsorbent. The objective was to assess the capability of the adsorbent to remove 100 mg/l of Alizarin spiked in all four water samples. To eliminate suspended materials from the samples, they were run through a 0.45 μm filter. The removal efficiency is observed to be greater than 94% as shown in Fig. 19.

It is worth noting that sewage and industrial water have a slightly lower removal efficiency because of the presence of competing ions, which are easily adsorbed onto the active sites.

### Reusability studies

**Fig. 20:**
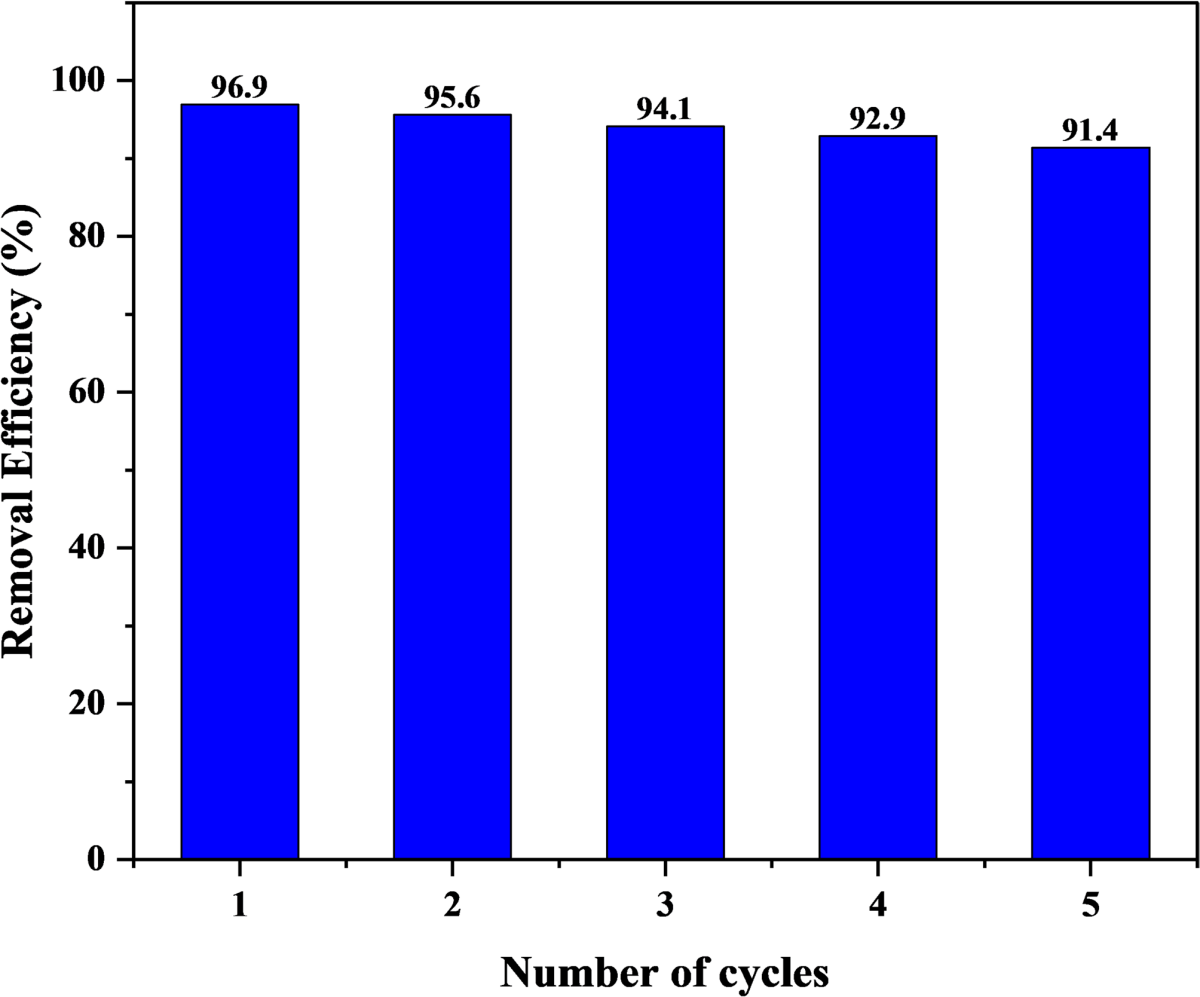
Reusability of the banana peel activated carbon for ALZ

The study assessed the reusability of the banana peel activated carbon to give an insight into the mechanistic approach, practical application of the study and economic feasibility. The reusability studies of Alizarin were carried out at optimal conditions, which involved five cycles. Desorption tests were carried out using a 0.1 M NaOH solution. During the first five cycles, the banana peel activated carbon demonstrated excellent reusability and stability, after which the removal efficiency reduced as shown in Fig. 20.

It is observed that the ALZ removal efficiency (%) for the 1st, 2nd, 3rd, 4th, and 5th cycles was 96.9, 95.6, 94.1, 92.9, and 91.45% respectively. This reduction might be attributed to the strong interaction between the adsorbate and adsorbent, where the active sites are occupied with ALZ even after desorption with NaOH.

### Comparison of BP-AC with other adsorbents

A comparative analysis between this study and different studies reported in literature by other authors who employed agricultural waste in the removal of alizarin from water is shown in Table 13. The comparison was based on the adsorption capacity, q_max_ (mg/g) of the adsorbent.

**Table 13:** Comparative study of literature on the removal of ALZ

Adsorbents	q_max_ (mg/g)	References
BP-Activated carbon	18.68	This work
Pine cone activated carbon	118.06	[51]
Dalbergia Sissoo activated carbon	0.47	[52]
Papaya leaves activated carbon	410.00	[53]
Alhagi maurorum activated carbon	8.20	[54]

Table 13 shows that the results obtained from this study using banana peel-activated carbon can be compared to previous work from literature that used different adsorbents. Therefore, it is observed to have obtained an adsorption capacity of 18.68 mg/g in less than 10 min. It is therefore concluded that based on the available data, BP-AC is a good adsorbent for ALZ removal from water.

## Conclusion

This study successfully demonstrated the efficacy of banana peel-activated carbon in the removal of Alizarin from water. Central composite design and Artificial neural networks proved to be powerful tools for modeling and optimizing the ALZ removal process. The quadratic model derived from CCD analysis accurately predicted removal efficiency, showing a statistically significant fit with an R² value of 0.9740 and an adequate precision of 27.5510. This model highlighted the significant individual effects of time, pH, and dosage, as well as the interaction between time and pH, and dosage and concentration. The optimization process identified the optimal conditions for maximizing Alizarin removal as: Time: 3.054 min, pH: 5.554, Dosage: 0.014 g, Concentration: 21.502 ppm. Under these conditions, a remarkable 92.181% removal was achieved, with a desirability function of 1, strongly validating the reliability and applicability of the RSM model. The study from the artificial neural networks also confirmed that the optimized parameters correlated strongly with each other, with a predicted removal efficiency of 96.26%. The adsorption mechanisms were investigated using kinetic studies, and it was concluded that the process occurs through chemisorption. This study also determined the mode of adsorption through the isotherms, and it was observed that it occurs mainly on multilayer surfaces.

## **Future** perspectives

The integration of RSM and ANN provides a robust framework for understanding complex adsorption processes and optimizing their parameters. This research contributes valuable insights into efficient wastewater treatment strategies using activated carbon. Future studies could explore the application of hybrid artificial intelligence models, which would give a much bigger correlation for the experimental and predicted data. It is also recommended that molecular simulations be investigated to further determine the interactions between the adsorbate and adsorbent. In addition, the preparation of different composites for the functionalized activated carbon would better enhance the removal efficiency.

## Data Availability

The datasets used and/or analyzed during the current study are available from the corresponding author on reasonable request.

## References

[1] Cheng F, Cheng P, Xie S, Wang H, Tang Y, Liu Y, Xiao Z, Zhang G, Yuan G, Wang K. Epidemiological trends and age-period-cohort effects on ischemic stroke burden across the BRICS-plus from 1992 to 2021. BMC Public Health. 2025;25:137. 39806358 10.1186/s12889-025-21310-9 PMC11731538

[2] Saxena V. Water quality, air pollution, and climate change: investigating the environmental impacts of industrialization and urbanization. Water Air Soil Pollut. 2025;236:73.

[3] Singh CK, Sodhi KK, Rajagopalan VN, Sharma V, Kaur S, Kumar J. Managing the complexity of emerging contaminants in aquatic environments: exploring their ecotoxicological impacts, detection techniques, and the use of innovative technologies for their remediation. Discover Catal. 2025;2:9.

[4] Hadibarata T. Assessing groundwater contamination from personal care products in malaysia: Sources, Mechanisms, and remediation strategies. Environ Qual Manage. 35, e70118 (2025).

[5] Barik D, Rakhi Mol KM, Anand G, Nandamol PS, Das D, Porel M. Environmental pollutants such as endocrine disruptors/pesticides/reactive dyes and inorganic toxic compounds metals, radionuclides, and metalloids and their impact on the ecosystem. Biotechnology for environmental sustainability. Springer; 2025. pp. 391–442.

[6] Elumalai P, Gao X, Parthipan P, Luo J, Cui J. Agrochemical pollution: A serious threat to environmental health. Curr Opin Environ Sci Health 100597 (2025).

[7] Weber R, Girones L, Förstner U, Tysklind M, Laner D, Hollert H, Forter M, Vijgen J. Review on the need for inventories and management of reservoirs of pops and other persistent, bioaccumulating and toxic substances (PBTs) in the face of climate change. Environ Sci Eur. 2025;37:48.

[8] Imamvali S, Prakash K, Bansal S, Tupakula S, Suresh AK, Al-Gburi AJA, Faruque MRI, Al-mugren KS. Label-free biosensing of persistent organic pollutants in sewage water using spoof surface plasmon polaritons. Sens Actuators Phys. 2025;388:116504.

[9] Gao Q, Sheng Q, Zhang S, Tang Y. Recent trends on MIL-88 (Fe) metal–organic frameworks: synthesis and applications in pollutant removal and detection. RSC Adv. 2025;15:26184–200. 40697458 10.1039/d5ra03049h PMC12282656

[10] Oguanobi NC, Aniagor CO, Okoronkwo G, Ude CN, Onu CE, Anike EN. Industrial dye effluent sources, generation, and value-added products. In: Engineered Biocomposites for Dye Adsorption. pp. 1–10. Elsevier (2025).

[11] Beg M, Saju J, Alcock KM, Mavelil AT, Markapudi PR, Yu H, Manjakkal L. Biodegradable biopolymers for electrochemical energy storage devices in a circular economy. RSC Sustain. 2025;3:37–63.

[12] Abdelhamid HN. Degradable Biopolymer-Based nanocomposites in water treatment and air purification: A review. ACS Appl Polym Mater. (2025).

[13] Bbumba S, Ssekatawa J, Karume I, Tebandeke E, Kigozi M, Yiga S, Setekera R, Ssebuliba J, Sekitto S, Mbabazi R. Prediction and optimization of Rhodamine B removal from water using metal-organic frameworks: RSM-CCD, ANN, non-linear kinetics, and isotherm studies. BMC Chem. 2025;19:1–20. 40696432 10.1186/s13065-025-01590-3 PMC12281964

[14] Bbumba S, Kigozi M, Karume I, Nsamba HK, Arum CT, Kiganda I, Maximillian K, Nazziwa RA, Ssekatawa J, Yikii CL. Enhanced photocatalytic degradation of methylene blue and Methyl orange dyes via transition Metal-Doped titanium dioxide nanoparticles. Asian J Chem Sci. 2024;14:17–41.

[15] Haleem A, Ullah M, Shah A, Farooq M, Saeed T, Ullah I, Li H. In-depth photocatalytic degradation mechanism of the extensively used dyes malachite green, methylene blue, congo red, and Rhodamine B via covalent organic framework-based photocatalysts. Water (Basel). 2024;16:1588.

[16] Anoua R, Touhtouh S, Rkhis M, Jouad E, Hajjaji M, Belhora A, Bakasse F, Sahraoui M, Płóciennik B, Zawadzka P. Optical and electronic properties of the natural Alizarin dye: theoretical and experimental investigations for DSSCs application. Opt Mater (Amst). 2022;127:112113.

[17] Mohamadpour F, Mohamadpour F. Photodegradation of six selected antipsychiatric drugs; carbamazepine, sertraline, amisulpride, amitriptyline, diazepam, and Alprazolam in environment: efficiency, pathway, and mechanism—a review. Sustainable Environ Res. 2024;34:8.

[18] Mansoor S, Manzoor S, Gull R, Gani G, Wani OA, Farooq S, Popescu SM, Arya VM, Park W-P, Chung YS. Biochar mediated remediation of emerging inorganic pollutants and their toxicological effects on plant and soil health. J Soil Sci Plant Nutr. 2025;25:1612–42.

[19] Sui Q, Yang X, Sun X, Zhu L, Zhao X, Feng Z, Xia B, Qu K. Bioaccumulation of polycyclic aromatic hydrocarbons and their human health risks depend on the characteristics of microplastics in marine organisms of Sanggou Bay, China. J Hazard Mater. 2024;473:134622. 38795479 10.1016/j.jhazmat.2024.134622

[20] Thakur R, Joshi V, Sahoo GC, Jindal N, Tiwari RR, Rana S. Review of mechanisms and impacts of nanoplastic toxicity in aquatic organisms and potential impacts on human health. Toxicol Rep 102013 (2025). 10.1016/j.toxrep.2025.102013 PMC11995781 40230517

[21] Mahmoud MA, Alsehli BR, Alotaibi MT, Hosni M, Shahat A. A comprehensive review on the application of semiconducting materials in the degradation of effluents and water splitting. Environ Sci Pollut Res. 2024;31:3466–94. 10.1007/s11356-023-31353-3 PMC10794432 38141122

[22] Arumugam B, Amanulla B, Venkatesh K, Sameem MS, Chang P-L, Ramaraj SK. Zinc oxide integrated carbon nitride nanocomposite for electrochemical sensing of catechol and photocatalytic degradation of Rhodamine B. Surf Interfaces 107009 (2025).

[23] Karume I, Bbumba S, Tewolde S, Mukasa I, harq ZT, Ntale M. Impact of carbonization conditions and adsorbate nature on the performance of activated carbon in water treatment. BMC Chem. 2023;17:162. 37993910 10.1186/s13065-023-01091-1 PMC10666421

[24] Karume I, Bbumba S, Kigozi M, Nabatanzi A, Mukasa I, harq ZT, Yiga S. One-pot removal of pharmaceuticals and toxic heavy metals from water using xerogel-immobilized quartz/banana peels-activated carbon. Green Chem Lett Rev. 2023;16:2238726.

[25] Rathinavel N, Veleeswaran A, Rathinam Y, Alagarsamy A. Turning waste into watt: usage of natural biomass activated carbon-based anode and septic tank wastewater for microbial fuel cell (MFC) based electricity generation. Carbon Trends. 2025;19:100461.

[26] Fonseca-Bermudez OJ, Giraldo L, Sierra-Ramirez R, Serafin J, Dziejarski B, Bonillo MG, Farid G, Moreno-Pirajan JC. Cashew nut shell biomass: a source for high-performance CO2/CH4 adsorption in activated carbon. J CO2 Utilization. 2024;83:102799.

[27] Moses K, Karume I, Bbumba S, Parvathalu K, Kasozi G, Tebandeke E. None-emission carbon nanomaterial derived from polystyrene plastic waste for the adsorption of carbon dioxide. Results Mater. 2025;100671. 10.1016/J.RINMA.2025.100671.

[28] Attia NF, Shaltout SM, Salem IA, Zaki AB, El-Sadek MH, Salem MA. Sustainable and smart hybrid nanoporous adsorbent derived biomass as efficient adsorbent for cleaning of wastewater from Alizarin red dye. Biomass Convers Biorefin. 2024;14:4989–5004.

[29] Salatein NM, Shaaban M, Fahim IS. Comparing low-cost activated carbon made from coffee waste and Bagasse to remove heavy metals and methylene blue dye. Results Chem. 2025;13:102020. 10.1016/J.RECHEM.2025.102020.

[30] Yikii CL, Bbumba S, Tebandeke E, Kigozi M, Karume I, Naziriwo B, Nyakairu GW, Yiga S, Ssekatawa J, Talibawo J. Application of carbon dioxide as a soft oxidant and promoter in metal-catalyzed oxidation reactions. Discover Catal. 2025;2:15.

[31] Aktar J. Batch adsorption process in water treatment. In: Intelligent environmental data monitoring for pollution management. pp. 1–24. Elsevier (2021).

[32] Turan AZ, Turan M. Removal of heavy metals and dyes from wastewaters by Raw and activated carbon hazelnut shells. Progress in nanoscale and Low-Dimensional materials and devices: Properties, Synthesis, Characterization, modelling and applications. Springer; 2022. pp. 907–33.

[33] Bbumba S, Karume I, Nsamba HK, Kigozi M, Kato M. An insight into isotherm models in physical characterization of adsorption studies. Eur J Appl Sciences–Vol. 12, (2024).

[34] Esmaili Z, Barikbin B, Shams M, Alidadi H, Al-Musawi TJ, Bonyadi Z. Biosorption of metronidazole using spirulina platensis microalgae: process modeling, kinetic, thermodynamic, and isotherm studies. Appl Water Sci. 2023;13:63.

[35] Yusop MFM, Abdullah AZ, Ahmad MA. Amoxicillin adsorption from aqueous solution by Cu (II) modified lemon Peel based activated carbon: mass transfer simulation, surface area prediction and F-test on isotherm and kinetic models. Powder Technol. 2024;438:119589.

[36] Butyrskaya E. Understanding the mechanism of monolayer adsorption from isotherm. Adsorption. 2024;30:1395–406.

[37] Moses K, Karume I, Bbumba S, Parvathalu K, Kasozi G, Tebandeke E. None-emission carbon nanomaterial derived from polystyrene plastic waste for the adsorption of carbon dioxide. Results Mater 100671 (2025).

[38] Fadl MG. Prediction of heavy metal biosorption mechanism through studying isotherm kinetic equations. Sci Rep. 2023;13:1576. 36709363 10.1038/s41598-023-28655-4 PMC9884289

[39] Kiani D, Wachs IE. Practical considerations for Understanding surface reaction mechanisms involved in heterogeneous catalysis. ACS Catal. 2024;14:16770–84. 39569155 10.1021/acscatal.4c05188 PMC11574757

[40] Masinga T, Moyo M, Pakade VE. Removal of hexavalent chromium by polyethyleneimine impregnated activated carbon: Intra-particle diffusion, kinetics and isotherms. J Mater Res Technol. 2022;18:1333–44.

[41] Benis KZ, Soltan J, McPhedran KN. Electrochemically modified adsorbents for treatment of aqueous arsenic: pore diffusion in modified biomass vs. biochar. Chem Eng J. 2021;423:130061.

[42] Bbumba S, Kigozi M, Nabatanzi J, Karume I, Arum CT, Nsamba HK, Kiganda I, Murungi M, Ssekatawa J, Nazziwa RA. Response Surface Methodology: A Review on Optimization of Adsorption Studies.

[43] Bbumba S, Kigozi M, Karume I, Nsamba HK, Arum CT, Kiganda I, Maximillian K, Nazziwa RA, Ssekatawa J, Yikii CL. Enhanced Photocatalytic Degradation of Methylene Blue and Methyl Orange Dyes via Transition Metal-Doped Titanium Dioxide Nanoparticles.

[44] Bbumba S, Kigozi M, Karume I, Arum CT, Murungi M, Babirye PM, Kirabo S. Prediction and optimization of process parameters using artificial intelligence and machine learning models. Asian J Appl Chem Res. 2025;16:11–33.

[45] Kigozi M, Koech RK, Orisekeh K, Kali R, Kamoga OLM, Padya B, Bello A, Kasozi GN, Jain PK, Kirabira JB. Cobs Porous Carbon-Based Materials With High Energy And Excellent Cycle Stability For Supercapacitor Applications. (2021).

[46] Oladipo AA, Ahaka EO, Gazi M. High adsorptive potential of calcined magnetic Biochar derived from banana peels for Cu2+, Hg2+, and Zn2 + ions removal in single and ternary systems. Environ Sci Pollut Res. 2019;26:31887–99. 10.1007/s11356-019-06321-5 31512127

[47] Shoaib AGM, Yılmaz M, El Sikaily A, Hassaan MA, El-Nemr MA, El Nemr A. Isotherm, kinetics and ANN analysis of methylene blue adsorption onto nitrogen doped Ulva lactuca Biochar. Sci Rep. 2025;15:10642. 40148409 10.1038/s41598-025-92973-y PMC11950198

[48] Ng KC, Burhan M, Shahzad MW, Ismail A, Bin. A universal isotherm model to capture adsorption uptake and energy distribution of porous heterogeneous surface. Sci Rep. 2017;7:10634. 28878385 10.1038/s41598-017-11156-6 PMC5587542

[49] Mia M. Mathematical modeling and optimization of MQL assisted end milling characteristics based on RSM and Taguchi method. Measurement. 2018;121:249–60. 10.1016/J.MEASUREMENT.2018.02.017.

[50] Bennett ND, Croke BFW, Guariso G, Guillaume JHA, Hamilton SH, Jakeman AJ, Marsili-Libelli S, Newham LTH, Norton JP, Perrin C, Pierce SA, Robson B, Seppelt R, Voinov AA, Fath BD, Andreassian V. Characterising performance of environmental models. Environ Model Softw. 2013;40:1–20. 10.1016/J.ENVSOFT.2012.09.011.

[51] Bhomick PC, Supong A, Baruah M, Pongener C, Sinha D. Pine cone biomass as an efficient precursor for the synthesis of activated biocarbon for adsorption of anionic dye from aqueous solution: Isotherm, kinetic, thermodynamic and regeneration studies. Sustain Chem Pharm. 2018;10:41–9. 10.1016/J.SCP.2018.09.001.

[52] Nawaz S, Salman SM, Ali A, Ali B, Shah SN, Rahman LU. Kinetics and thermodynamics investigations of efficient and eco-friendly removal of Alizarin red S from water via acid-activated dalbergia Sissoo leaf powder and its magnetic iron oxide nanocomposite. Front Chem. 2024;12:1457265. 39385963 10.3389/fchem.2024.1457265 PMC11462623

[53] Moheb M, El-Wakil AM, Awad FS. Highly porous activated carbon derived from the Papaya plant (stems and leaves) for superior adsorption of Alizarin red s and methylene blue dyes from wastewater. RSC Adv. 2025;15:674–87. 39781019 10.1039/d4ra07957d PMC11708045

[54] Akram B, Umar A, Ali MA, Elshikh MS, Igwegbe CA, Iqbal R, Ghosh S. Kinetic and thermodynamic analysis of Alizarin red S biosorption by alhagi maurorum: a sustainable approach for water treatment. BMC Biotechnol. 2024;24:85. 39478538 10.1186/s12896-024-00913-x PMC11523905

